# The Influence of Data Resolution on Predicted Distribution and Estimates of Extent of Current Protection of Three ‘Listed’ Deep-Sea Habitats

**DOI:** 10.1371/journal.pone.0140061

**Published:** 2015-10-23

**Authors:** Lauren K. Ross, Rebecca E. Ross, Heather A. Stewart, Kerry L. Howell

**Affiliations:** 1 Marine Biology & Ecology Research Centre, Marine Institute, Plymouth University, Plymouth, United Kingdom; 2 British Geological Survey, Murchison House, West Mains Road, Edinburgh, United Kingdom; University of Sydney, AUSTRALIA

## Abstract

Modelling approaches have the potential to significantly contribute to the spatial management of the deep-sea ecosystem in a cost effective manner. However, we currently have little understanding of the accuracy of such models, developed using limited data, of varying resolution. The aim of this study was to investigate the performance of predictive models constructed using non-simulated (real world) data of different resolution. Predicted distribution maps for three deep-sea habitats were constructed using MaxEnt modelling methods using high resolution multibeam bathymetric data and associated terrain derived variables as predictors. Model performance was evaluated using repeated 75/25 training/test data partitions using AUC and threshold-dependent assessment methods. The overall extent and distribution of each habitat, and the percentage contained within an existing MPA network were quantified and compared to results from low resolution GEBCO models. Predicted spatial extent for scleractinian coral reef and *Syringammina fragilissima* aggregations decreased with an increase in model resolution, whereas *Pheronema carpenteri* total suitable area increased. Distinct differences in predicted habitat distribution were observed for all three habitats. Estimates of habitat extent contained within the MPA network all increased when modelled at fine scale. High resolution models performed better than low resolution models according to threshold-dependent evaluation. We recommend the use of high resolution multibeam bathymetry data over low resolution bathymetry data for use in modelling approaches. We do not recommend the use of predictive models to produce absolute values of habitat extent, but likely areas of suitable habitat. Assessments of MPA network effectiveness based on calculations of percentage area protection (policy driven conservation targets) from low resolution models are likely to be fit for purpose.

## Introduction

Limited spatial location data for vulnerable species and habitats is thought to be the most common limitation to progress in the designation of protected areas for the conservation of such species and habitats [1]. Habitat suitability modelling (HSM) provides a means to produce full coverage estimated spatial data where valuable species distribution information is lacking. The resulting predictions may be used to support marine conservation management decisions. Political initiatives often set percentage conservation targets by which the success (or otherwise) of these decisions in the protection of vulnerable marine ecosystems (VMEs) is measured. To evaluate progression toward these targets and understand how much of a habitat is protected by the conservation management strategy in place, reliable habitat location data are again a crucial pre-requisite.

The principle of HSM is in formalizing the relationship between environmental drivers and species' distributions [2]. Bathymetric data provides a surrogate for the combined influence of several environmental parameters such as temperature, pressure, current speed, direction of flow, food availability and sediment type on deep-sea benthic biological community structure [3–4]. Terrain features derived from bathymetry data therefore can act as useful predictor variables for HSM of deep-sea benthic communities [5], where continuous environmental data are often lacking.

Characterisation of the seabed in terms of terrain parameters is highly scale dependent [6–9]. The convergence of extreme terrain attribute values toward the means with the lowering of data resolution [9] is thought to result in a loss in predictive power when applied to HSM. Guisan & Thuiller [10] stressed the importance of correct spatial matching between presence data and environmental data to avoid artificial expansion of a species’ preferred conditions. Davies *et al* [11] used HSM to predict the global distribution of *Lophelia pertusa* (Linnaeus, 1758). The 1° x 1° temperature grid failed to represent abrupt changes in water temperature, leading to predicted presence outside the species’ normal thermal tolerance limit, highlighting the importance of correct spatial matching.

The habitat distribution of deep-sea fauna exhibits patterns of variability on fine spatial scales [12]. Certain fauna (e.g., cold-water corals) have also shown strong associations with topographic features on much larger spatial scales (e.g., seamounts and carbonate mounds) [13], leading to the question of what scale these habitats should be modelled? However, use of low resolution bathymetry data as a predictor can mask fine scale topographic features known to support high levels of biodiversity such as small carbonate mounds, iceberg plough-marks and small scours on the seabed [11, 14–15] something that has resulted in the failure of previous HSMs to predict known habitat presence [11, 14–16]. Studies suggest models built from bathymetric data of a higher resolution [9, 15, 17], or inclusive of multi-resolution terrain attributes [18] would more accurately predict habitat suitability in these areas of high predictive error.

Predictions based on low resolution bathymetry data are likely to overestimate habitat spatial extent [12, 15]. Gorgonian species distribution modelled using bathymetric data from the General Bathymetric Chart of the Oceans (GEBCO) (750m) resulted in a spatial extent on Hatton Bank twice the size of that produced from high resolution (50m) data [17]. The percentage of habitat protected within a marine protected area (MPA) network may not necessarily be affected by model resolution in the same way, or at all.

The influence that data resolution has on the accuracy of HSM and management effectiveness in the deep-sea environment has received inadequate attention, yet it is important to the development of marine conservation strategy and MPA assessment. This study builds on the work of Marshall [17], Ross and Howell [15] and Rengstorf *et al*. [9, 12,19]. We focus on building high resolution HSMs and present the distributions of suitable habitat areas for three deep-sea habitats, all considered as VMEs under United Nations General Assembly Resolution (UNGA) 61/105: scleractinian (Vaughan & Wells, 1943) cold-water coral reefs (SclerReef) (comprised of *L*. *pertusa* and / or *Solenosmilia variabilis* (Duncan, 1873) reefs), *Pheronema carpenteri* (Wyville-Thomson, 1869) aggregations (PcAggs), and *Syringammina fragilissima* (Brady, 1883) aggregations (SfAggs).

*L*. *pertusa* and *S*. *variablis* are two of the most prominent reef-building species of scleractinian coral known to form large densely branched colonies in the NE Atlantic deep-sea [20–21]. *L*. *pertusa* reefs comprise the majority of the dataset and are mostly found attached to hard substrata between 200m and 400m water depth [11] in areas of strong current flow [11, 22] associated with steep slopes and topographic peaks such as seamounts. Within the study area *S*. *variabilis* occurs at depths between ~800m and 2165m, often with a diverse associated benthic community [20–21]. *P*. *carpenteri* is a deep-sea glass sponge that forms aggregations within a narrow environmental niche. In the study area they occupy a depth range of between 1000m and 1300m [23], on fine-grained sediment bottoms [24] in areas of high productivity with enhanced current flow [25]. *S*. *fragilissima* is a unique and large unicellular organism found exclusively in the deep sea. It is one of the most commonly observed species of Xenophyophore in the NE Atlantic [26]. Dense aggregations form under nutrient rich conditions on fine-grained sediment slopes, or near topographically distinct features [27].

This study investigates the following hypotheses:

1. High resolution models perform better than low resolution models, in terms of the assessment methods described in this paper.
2. Estimates of predicted habitat extent decrease with increasing data resolution.
3. Estimates of predicted habitat distribution contract around areas of predicted high suitability with increasing data resolution.
4. Estimates of percentage area protected within an MPA network remain similar between high and low resolution models.

## Methods

### Site description

**Fig 1:**
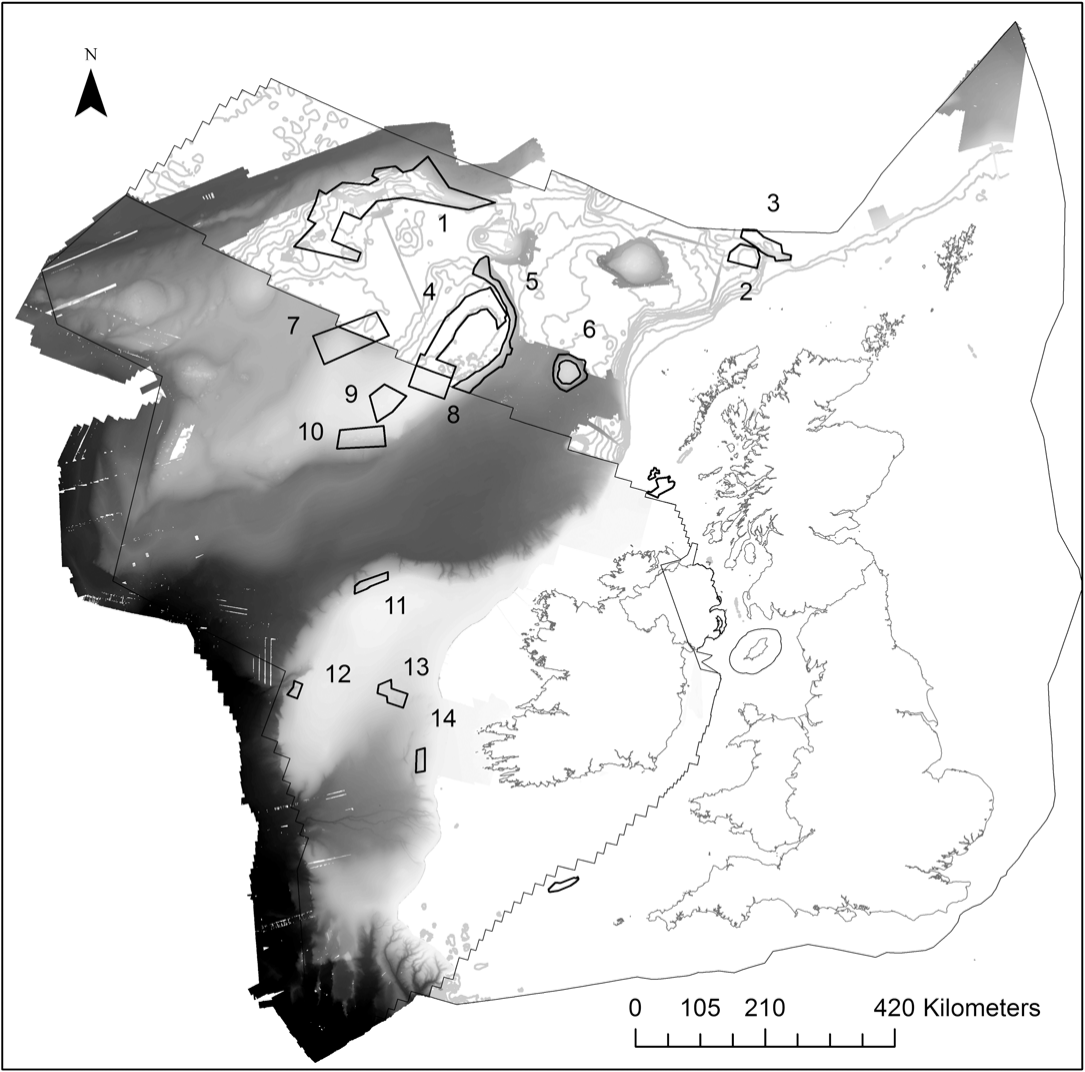
Study area and location of MPAs. Black outlines mark the borders of UK and Irish waters. Continuous greyscale bathymetry marks the available high resolution data extent. MPAs considered in this study are outlined in black and numbered: 1 –Hatton Bank pSAC and NEAFC Closure, 2 –Darwin Mounds cSAC, 3 –Wyville Thompson Ridge cSAC, 4 –NW Rockall cSAC and NEAFC Closure, 5 –East Rockall Bank pSAC, 6 –Anton Dohrn pSAC, 7 –West Rockall NEAFC Closure, 8 –Haddock Box NEAFC Closure, 9 –SW Rockall (Empress of Britain Bank) NEAFC Closure, 10 –Logachev Mounds NEAFC Closure, 11 –NW Porcupine Bank cSAC, 12 –SW Porcupine Bank cSAC, 13 –Hovland Mound Province cSAC, 14 –Belgica Mound Province cSAC. Isobaths are every 200m from 200–3200m. Map projected in Albers Equal Area Conic with modified standard parallels (parallel 1 = 50.2, parallel 2 = 58.5).

The study considers the full extent of the Irish, and a partial extent of the UK’s extended continental shelf in the N E Atlantic, from the 200m contour along the shelf-edge to their western boundaries (Fig 1). A network comprising three different types of MPA exist in this area for the protection of deep-sea habitats identified as either threatened and declining habitats under the Annex V of the OSPAR Convention, or as VMEs under UNGA61/105.

### Biological data

Presence/absence datasets for all 3 VMEs were compiled from 222 video transects collected from several research cruises that took place in the study area over a period of six years ending in 2011 with additional presence/absence data for SclerReef and PcAggs obtained from records held at the National Oceanography Centre, Southampton, from trawling activities carried out in the Porcupine Seabight (PSB) and Porcupine Abyssal Plain (PAP) between 1977 and 2000 (See S1 File for details of data sources). Since the data were not all collected with the same gear type, and abundance estimates are not comparable between gear types, the data were considered as presence-absence data.

All biological data were identical to that used to build habitat suitability maps based on low resolution environmental data in Ross & Howell [15].

### Predictor variables

High-resolution multibeam bathymetry data (maximum cell size of 200x200m) was obtained from various sources (S1 File). Details of the multibeam systems used can be found in the corresponding references. Multibeam datasets were all re-projected into Albers Equal Area Conic with modified standard parallels (Parallel 1: 50.2°, Parallel 2: 58.5°), resampled at a cell size of 200x200m, and merged to produce a single bathymetry layer. Low resolution bathymetry data was obtained from the GEBCO 2008 30 arc-s grid and also re-projected into Albers Equal Area Conic with modified standard parallels (Parallel 1: 50.2°, Parallel 2: 58.5°) and a cell size of 750x750m.

Seven topographic variables were derived from both bathymetry layers. Slope, curvature, plan curvature, and profile curvature were created using the ArcGIS [28] Spatial Analyst extension. Rugosity, broad scale and fine scale bathymetric position index (BPI) were created using the Benthic Terrain Modeller extension [29]. BPI broad was calculated with an inner radius of 1 and an outer radius of 33 resulting in a scale factor of 24.75km using low resolution data and 6.6km using high resolution data. BPI fine was calculated with an inner radius of 1 and an outer radius of 3, resulting in a scale factor of 2.25km using low resolution data and 0.6km using high resolution data.

Further information on the specifics of using these variables as surrogates is available in existing literature [5, 15, 30–31].

### Modelling

Biological data was reduced to one point per cell of environmental data in ArcGIS. The video transects have a field of view that covers less than 10m and the trawl mouth opening was only 8m across. We therefore felt that absence data could not be considered reliable when used with environmental data cells of size 200x200m and / or 750x750m resolution. The existence of potential false absences within our dataset, a problem referred to as “imperfect detection” in Lahoz-Monfort et al [32], means that rather than estimating where species occur, we are only able to estimate where they are detected, an inherent limitation of the models.

Using Guillera-Arroita et al.’s, [33] simple framework that summarizes how interactions between data type and the sampling process (i.e. imperfect detection and sampling bias) determine the quantity that is estimated by a habitat suitability model, we assessed that we were able to model, at best, relative likelihood data using either a presence-absence or presence-background approach. We opted to use a presence-background modelling approach with the aim of being very clear about the data limitations. While relative likelihoods are not considered appropriate for use in determining area of occupancy [33], real world datasets on the scale at which we are modelling very rarely meet the conditions required to achieve probabilities rather than relative likelihoods. Our aim in this paper was to compare relative estimates of extent and distribution (a measure of area of occupancy) obtained from high and low resolution models rather than provide actual estimates of extent, and thus we feel the use is justified on this occasion.

Maximum entropy (MaxEnt) modelling [34] has been found to be one of the best performing presence-background modelling techniques [35] and was therefore employed to build the habitat suitability maps for this study. All 750m grids were aligned and cut to the 200 m cell size grids as required by MaxEnt. This process did not include any attempt to increase the resolution of the 750m data through the use of kriging or interpolation, since the purpose was merely to align the grids for use in the MaxEnt software.

Pre-selection of significant environmental variables was undertaken using both presence and absence data in a Generalised Additive Modelling (GAM) approach prior to MaxEnt modelling. Highly correlated variables were identified and the least significant correlate was removed from the analysis (see S2 File for details of correlate removal and GAMs). The final variables selected for each model is given in Table C in S2 File.

The Marine Geospatial Ecology Tools add-on [36] was used to extract terrain derived data from the locations of plotted presence and absence data points in ArcGIS. MaxEnt was run using the samples-with-data (SWD) approach using presence and absence data as background. This method of ‘target-group’ background sampling controls for sample bias, improves predictive performance [37], and allows the relative likelihood model output to be considered as proportional to the probability of occurrence [33]. All models were run with MaxEnt version 3.3.3. Preliminary runs trialled different regularization settings to reduce overfitting [37] and a regularization parameter of 3 was selected for all three models. The Maxent output is a logistic index of relative likelihood with values between 0 (low likelihood) and 1 (high likelihood). One master model was created for each listed habitat.

### Model evaluation

The full model dataset for each habitat was split into training (75%) and test (25%) datasets, a process that was repeated to build ten new partitioned datasets. Training and test datasets were compiled manually instead of using the MaxEnt replicates setting to control for spatial autocorrelation within transects [38]. The prevalence in each partition was then checked to be approximately equal to the full model dataset (±0.01). A new model was built with each new partition in R with the ‘dismo’ package version 0.8–11 [39] and MaxEnt Java program. Models were assessed using the presence/absence model evaluation library [40] in R [41] using both threshold-dependant and threshold independent approaches.

The area under the receiver operating characteristic curve (AUC) was calculated for each full model, and all training and test datasets for SclerReef, PcAggs, and SfAggs. Mean and standard deviation of AUC over the 10 training and test partitions was calculated. Although AUC is a widely used statistic in measuring the performance of HSM, it is not without criticism [42–44] and so the reliability of all models were also assessed using threshold-dependent model evaluation indices [45].

To transform the MaxEnt output from a logistic index of relative likelihood of suitable habitat to presences/absences three thresholding approaches termed ‘good’ by Liu *et al* [46] were used to first determine a threshold for each model. Three sensitivity-specificity combined methods including sensitivity-specificity equality (Sens = Spec) [47], sensitivity-specificity sum maximization (MaxSens+Spec) [48] and an approach based on the minimum distance to the top-left corner (0,1) in ROC plot (MinROCdist) [47] were applied. Model performances with each different thresholding method applied were assessed using three indices: sensitivity (Sens.), specificity (Spec.) and percent correctly classified (PCC) [45, 49]. Sensitivity equates to the proportion of the presence observations predicted correctly as presence, while specificity equates to the proportion of the absence observations that were correctly predicted as absences. PCC is the number of correctly classified observations (presence and absence) as a percentage of the total number of observations. Values were then classified on a five-point scale: excellent (1–0.9), good (0.9–0.8), fair (0.8–0.7), poor (0.7–0.6) and fail (0.6–0.5). Considering the averaged threshold-dependent metrics for the partitions together with full model metrics, a final threshold was chosen to maximize final model performance. Best model performance was determined as that which gave the highest score on average across all measured indices. Variable importance was evaluated using the jackknife plots and response curves from the final MaxEnt model output.

### Quantification of habitat distribution

MaxEnt output relative likelihood maps were transferred to ArcGIS as raster grids and masked for novel climates (combinations of environmental parameters not represented in the model input data). The maps were then thresholded into predicted presence/absence. Relative likelihoods that fell below the chosen threshold for each habitat were converted to a constant absence raster (cell value of 0); relative likelihoods above the threshold were retained to later differentiate between areas of high relative likelihood denoted as presence and low relative likelihood denoted as presence. A standard deviation of all presence likelihoods from the ten partitioned models was also calculated to create a confidence map for each habitat.

### High resolution vs. low resolution

Performance of high resolution models measured using AUC, sensitivity, specificity and PCC was compared with the values published in Ross & Howell [15] for equivalent low resolution models. Low resolution (750m) predicted presence / absence layers from Ross & Howell [15] and new high resolution (200m) presence likelihood rasters for each habitat were loaded into ArcGIS. Using the Spatial Analyst tool, both were clipped to cover exactly the same spatial extent with aligned grids of cell size 200m x 200m in order to facilitate comparison between models. The number of presence raster cells within MPA polygons were calculated and then expressed as percentages of total presences in the whole study area, UK waters, and Irish waters. Predicted distributions at both scales were visually compared for all habitats to identify major discrepancies/similarities in the final predicted distributions in support of testing hypothesis 3.

## Results

### Modelling

**Fig 2:**
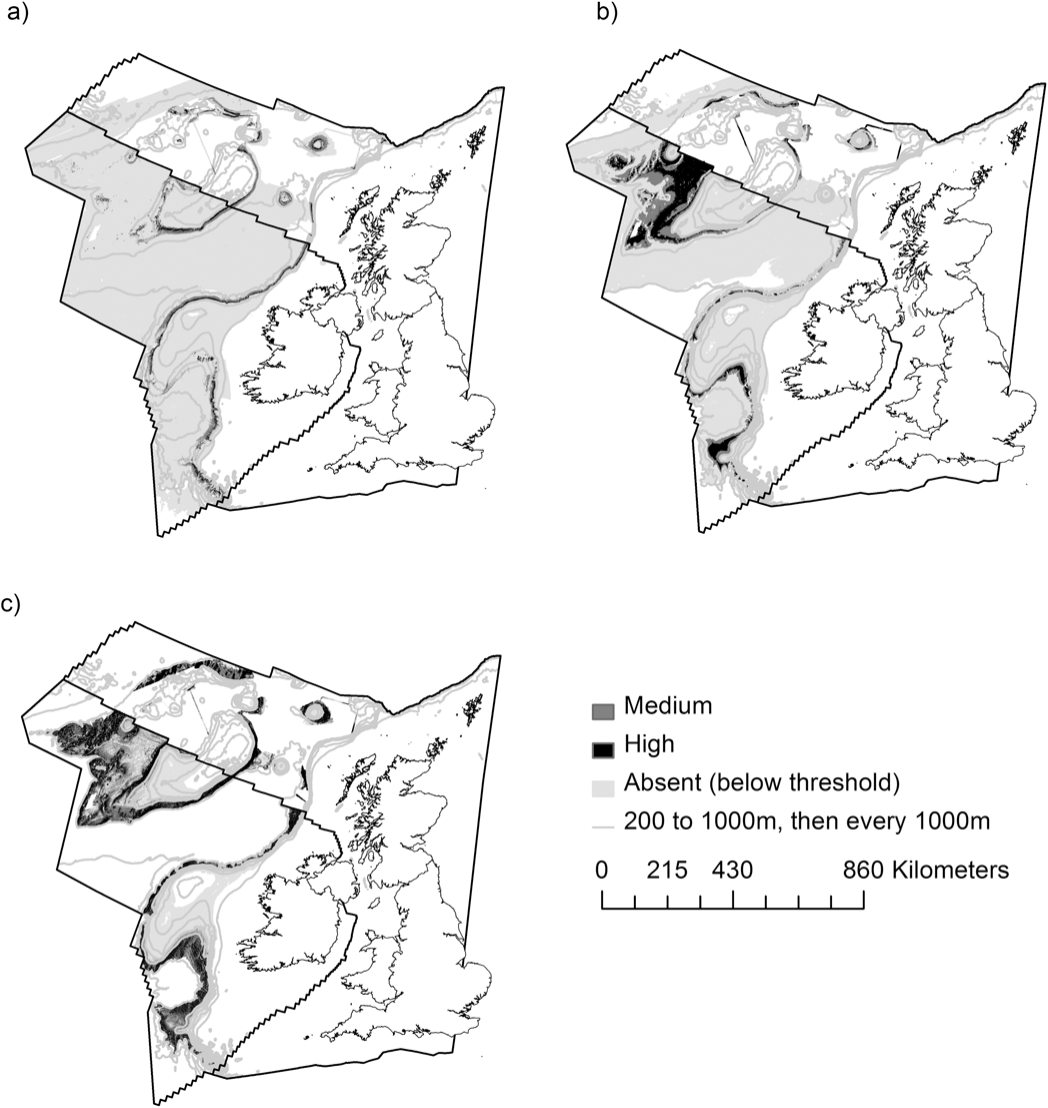
Full-model, habitat suitability prediction maps. a) scleractinian cold-water coral reef distribution; b) *Pheronema carpenteri* aggregation distribution; c) *Syringammina fragilissima* aggregation distribution. Threshold values for each habitat are as follows: a) threshold 0.43; b) threshold 0.34; c) threshold 0.41. For all three VMEs, the boundary between medium and high relative likelihood of suitable habitat is 0.55. Where white background is visible, prediction has been masked because of novel climates. Maps projected in Albers Equal Area Conic with modified standard parallels (parallel 1 = 50.2°, parallel 2 = 58.5°).

Final habitat suitability maps for SclerReef, PcAggs and SfAggs are presented in Fig 2.

### Model evaluation

After consideration of performance indices (PCC, Sens. and Spec.) for all models (Table 1), MinROCdist was selected as the thresholding method to be used for all models resulting in the thresholds 0.43 (SclerReef), 0.34 (PcAggs), and 0.41 (SfAggs), (note that for SclerReef MaxSens+Spec and MinROCdist recommended the same threshold).

**Table 1:** Threshold-dependent evaluation indices for training, test, and full models.

	Average Training			Average Test			Full Model			
Thresholding approach	PCC (SD)	Sens. (SD)	Spec. (SD)	PCC (SD)	Sens. (SD)	Spec. (SD)	PCC	Sens.	Spec.	Threshold
a)										
Sens = Spec	0.83 (0.01)	0.83 (0.04)	0.83 (0.01)	0.77 (0.02)	0.77 (0.02)	0.77 (0.02)	0.85	0.85	0.84	0.42
MaxSens+Spec	0.79 (0.01)	0.90 (0.03)	0.78 (0.01)	0.76 (0.02)	0.76 (0.02)	0.75 (0.02)	0.85	0.85	0.85	0.43
MinROCdist	0.83 (0.01)	0.85 (0.04)	0.83 (0.01)	0.79 (0.02)	0.79 (0.02)	0.79 (0.02)	0.85	0.85	0.85	0.43
b)										
Sens = Spec	0.95 (0.01)	0.95 (0.03)	0.95 (0.01)	0.95 (0.01)	0.95 (0.04)	0.95 (0.01)	0.96	0.96	0.96	0.33
MaxSens+Spec	0.95 (0.01)	0.98 (0.02)	0.95 (0.01)	0.95 (0.01)	1.00 (0.00)	0.94 (0.01)	0.94	0.99	0.94	0.24
MinROCdist	0.95 (0.01)	0.98 (0.02)	0.95 (0.01)	0.95 (0.01)	0.99 (0.01)	0.95 (0.01)	0.96	0.96	0.96	0.34
c)										
Sens = Spec	0.86 (0.01)	0.86 (0.04)	0.86 (0.01)	0.79 (0.03)	0.79 (0.03)	0.79 (0.03)	0.87	0.87	0.87	0.43
MaxSens+Spec	0.83 (0.01)	0.94 (0.03)	0.82 (0.02)	0.74 (0.03)	0.98 (0.02)	0.72 (0.03)	0.82	0.99	0.8	0.27
MinROCdist	0.87 (0.01)	0.88 (0.04)	0.87 (0.01)	0.79 (0.03)	0.89 (0.06)	0.78 (0.03)	0.86	0.9	0.86	0.41

Threshold-dependent evaluation indices for

a) scleractinian cold-water coral reef

b) *Pheronema carpenteri* aggregations

c) *Syringammina fragilissima* aggregations. Evaluation metrics are: per cent correctly classified (PCC), sensitivity (Sens.) and specificity (Spec.) Training and test model indices are given as average evaluation scores calculated from the ten partition models for each habitat, including standard deviations (SD).

The SclerReef full model and average training partition AUC values (Table 2) were both considered excellent (1–0.9), while average test partition AUC was considered good (0.9–0.8). The threshold determined by MinROCdist yielded good (0.9–0.8) results for full model PCC, sensitivity and specificity (Table 1). Training and test sensitivity were also good (0.9–0.8), with both training and test partitions resulting in fair (0.8–0.7) PCC and specificity. PcAggs full model, average training and test AUC scores were excellent (1–0.9). The threshold-dependent metrics were also considered excellent (1–0.9) for full model, training and test partitions. The SfAggs full model, average training and average test partition AUC values were considered excellent (1–0.9). After the chosen thresholding method was applied, all average training partition metrics were considered good (0.9–0.8), as were full model PCC, specificity and test sensitivity. Full model sensitivity was considered excellent (1–0.9), test PCC and specificity were considered to be fair (0.8–0.7).

**Table 2:** Final high and low resolution thresholds and associated evaluation metrics.

		Average Training				Average Test				Full Model			
Resolution	Threshold (approach)	AUC	PCC (SD)	Sens. (SD)	Spec. (SD)	AUC	PCC (SD)	Sens. (SD)	Spec. (SD)	AUC	PCC	Sens.	Spec.
a)													
High	0.43 (MinROCdist)	0.91	0.83 (0.01)	0.85 (0.04)	0.83 (0.01)	0.88	0.79 (0.02)	0.79 (0.02)	0.79 (0.02)	0.92	0.85	0.85	0.85
Low	0.48 (MinROCdist)	0.82	0.79 (0.01)	0.75 (0.05)	0.79 (0.02)	0.75	0.75 (0.03)	0.69 (0.09)	0.75 (0.03)	0.86	0.82	0.75	0.82
b)													
High	0.34 (MinROCdist)	0.94	0.95 (0.01)	0.98 (0.02)	0.95 (0.01)	0.96	0.95 (0.01)	0.99 (0.01)	0.95 (0.01)	0.99	0.96	0.96	0.96
Low	0.19 (MinROCdist)	0.99	0.96 (0.01)	0.98 (0.02)	0.95 (0.01)	0.99	0.96 (0.01)	0.96 (0.01)	0.96 (0.01)	0.99	0.95	0.96	0.95
c)													
High	0.41 (MinROCdist)	0.91	0.87 (0.01)	0.88 (0.04)	0.87 (0.01)	0.9	0.79 (0.03)	0.89 (0.06)	0.78 (0.03)	0.94	0.86	0.9	0.86
Low	0.31 (MinROCdist)	0.93	0.83 (0.02)	0.90 (0.06)	0.83 (0.02)	0.89	0.80 (0.03)	0.92 (0.06)	0.79 (0.03)	0.93	0.82	0.93	0.81

Final high and low resolution thresholds with associated threshold-dependent evaluation and AUC metrics for average training, average test and full models.

a) scleractinian cold-water coral reef

b) *Pheronema carpenteri* aggregations

c) *Syringammina fragilissima* aggregations. Evaluation metrics are per cent correctly classified (PCC), sensitivity (Sens.) and specificity (Spec.). Standard deviations (SD) given for evaluation indices for average training and test models (built from partitioned models 1–10).

### Assessment of variable importance

For all models the order of variables from greatest to smallest in terms of isolated model gain is provided in Table A in S3 File.

Examination of the jackknife plot for SclerReef full model revealed rugosity (200m) to be the most useful and informative variable when used in isolation, producing the highest model gain. The environmental variable that decreased gain the most when excluded from the model was bathymetry (200m), suggesting this variable holds the majority of the information used to model habitat suitability that is not represented by any other variable included in the full model. For both the PcAggs and SfAggs models bathymetry (200m) was the most useful predictor producing the greatest change in gain when excluded or used in isolation.

### High resolution vs. low resolution

#### Comparison of model performance

A two sample t-test on AUC data found that SclerReef full model performance significantly improved with the use of high resolution data (t = 5.6814, df = 9.558, p-value < 0.01) while PcAggs got worse (t = -4.3333, df = 9, p-value < 0.01) (All variances were non-equal so the Welch t-test was used in which the degrees of freedom are approximated using the Welch–Satterthwaite equation). However an increase in predictor variable data resolution resulted in no change in the performance of SfAggs model when assessed using AUC (t = 0.8361, df = 9.783, p-value = 0.42). Threshold-dependent evaluation of high resolution models suggested an overall improvement in performance when compared to low resolution model evaluation in Ross & Howell [15] (Table 2).

#### Comparison of predicted extent

**Fig 3:**
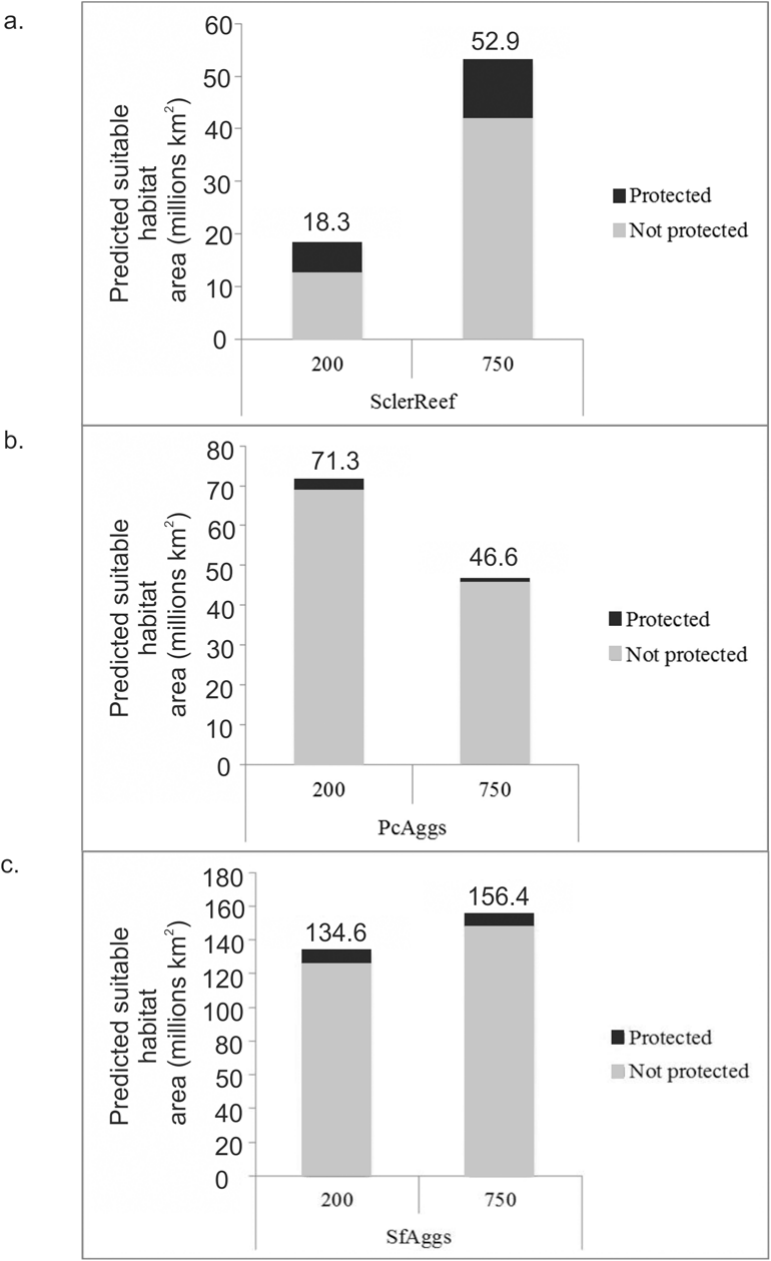
Total predicted suitable habitat extent. A comparison of predicted suitable habitat areas produced from full models using 200m bathymetric data and 750m bathymetric data. Data labels show total predicted area in millions km^2^. Scleractinian cold-water coral reef are represented by a), *Pheronema carpenteri* aggregations by b) and *Syringammina fragilissima* aggregations by c).

Spatial extent of all three habitats varied between the two data resolutions. SclerReef modelled using high resolution bathymetric data covered an area only 35% of that modelled using low resolution data (Fig 3A). PcAggs distribution on the other hand covers a greater area when modelled using high resolution data, covering an area 53% greater than that modelled using low resolution data (Fig 3B). SfAggs distribution is less prevalent when modelled using high resolution data, covering an area 83% the size of that when modelled using low resolution data (Fig 3C).

#### Comparison of predicted distribution

**Fig 4:**
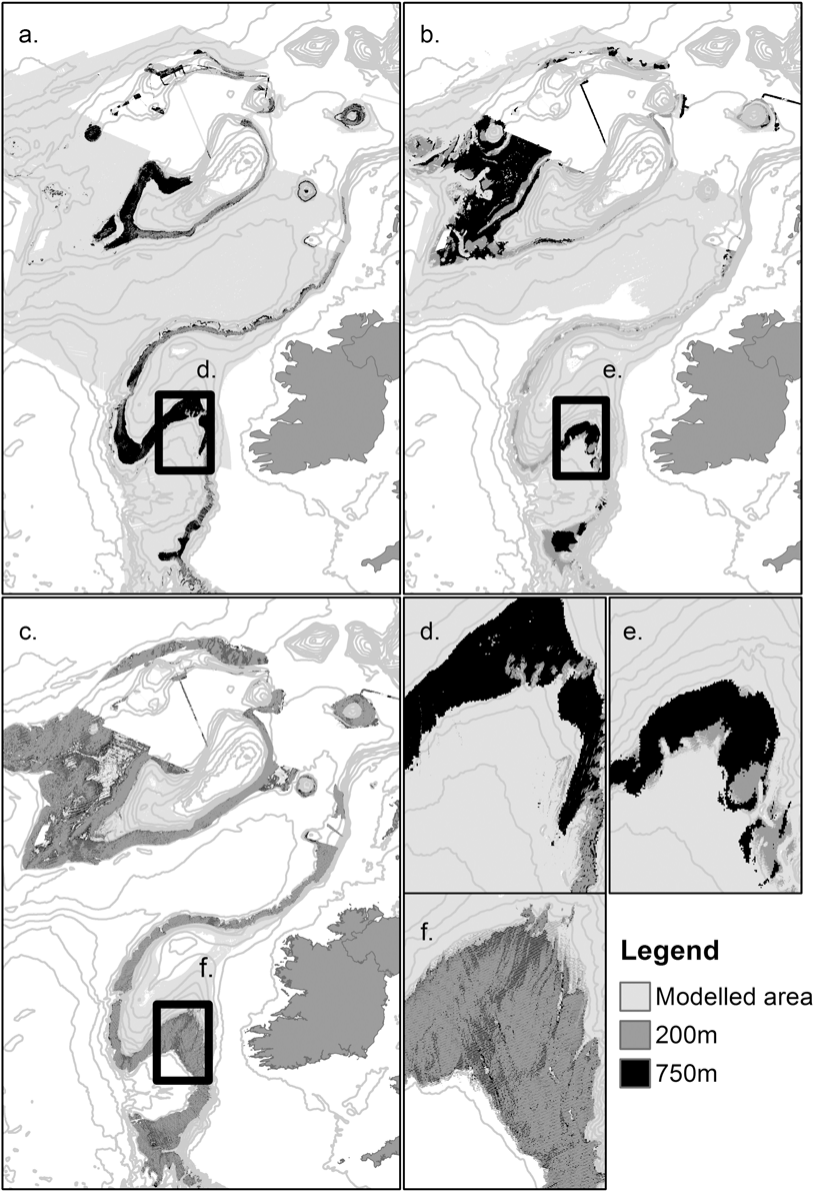
Predicted VME distribution maps and Porcupine Seabight detail. Full model predictions for high (200m) and low (750m) resolution models of a) scleractinian cold-water coral reef; b) *Pheronema carpenteri* aggregations; c) *Syringammina fragilissima* aggregations with insets d), e), and f), showing a zoomed area of the Porcupine Seabight. For each VME the model with the largest extent is displayed on top. Therefore the high resolution model is displayed on top of the low resolution model for a) and c); low is displayed on top of high for b).

For each VME, modelled spatial distribution throughout the study area of all three habitats varied between the two data resolutions (Fig 4). The distribution of SclerReef for example based on high resolution data produced distinctly different patterns along the continental shelf-edge, along the western slope of Rockall Bank and over the Anton Dohrn Seamount (ADS) (Fig 4) to that predicted in Ross & Howell [15]. The low resolution SclerReef model predicted almost the entire area contained within the Hovland Mound Province cSAC within the PSB as suitable habitat, whereas the high resolution model predicted presences on just the topographic peaks (Fig 4). Predicted habitat distribution did not therefore contract around areas of predicted high suitability with increasing data resolution as hypothesized.

#### Comparison of percentage area protected

Assessment of existing area closures for the protection of SclerReef, PcAggs and SfAggs (Table 3) revealed SclerReef suitable environments to be the best protected of all three habitats (29%). The level of SclerReef habitat protection decreased when calculated for Irish waters alone (17.6%). PcAggs are the least protected habitat, with only 2.9% of its predicted suitable environments contained within the current MPA network. 6% of SfAggs suitable environment lie within the MPA boundaries.

**Table 3:** Percentages of predicted suitable habitat area protected by the MPA network in place.

	a)		b)		c)	
	High	Low	High	Low	High	Low
Predicted presence in UK and Irish waters:						
In any MPA (cSAC, pSAC or NEAFC closures)	29	20.2	2.9	1.9	6	5.5
In NEAFC closures	13.7	12.3	1.7	1.2	2.6	2.4
Predicted presence in UK waters:						
In any UK MPA (cSAC, pSAC or NEAFC closures)	56.5	56	14.7	11	18.7	17.3
In UK cSACs	1.4	0.7	0	0	0.1	0.06
UK pSACs	54.7	55.2	13.3	10.9	17.8	16.4
Predicted presence Irish waters:						
In any Irish MPA (cSAC and NEAFC closures)	17.6	12.6	1.3	0.4	2.9	2.7
In Irish cSACs	8	4.6	0.1	0	0.8	0.7

Percentages of predicted suitable environments for each listed habitat currently protected within the wider MPA network, and by national jurisdiction, when modelled at both environmental resolutions (high = 200m, low = 750m).

a) scleractinian cold-water coral reef

b) *Pheronema carpenteri* aggregations

c) *Syringammina fragilissima* aggregations.

Percentage protection offered to SclerReef, PcAggs, and SfAggs by the existing MPA network were all larger using the habitat suitability models built with high resolution bathymetry data (Table 3), except for SclerReef within UK pSACs (54.7% of high resolution distribution, 55.2% of low resolution distribution).

## Discussion

### High resolution vs. low resolution

#### Comparison of model performance

Our study found performance varied between habitats, in consideration of the standard model assessment methods (AUC, sensitivity, specificity and PCC) used (Table 2), with improvement, deterioration and no change in performance observed between high and low resolution models for SclerReef, PcAggs, and SfAggs respectively. Similar inconsistency in the response of terrestrial model performance to a decrease in predictor variable resolution has also been observed [50–53]. The data resolutions used in this study (200 and 750m grid cell size) were selected on the basis that they reflect the data resolution currently available to support management within the deep sea. Previous studies that have considered the effect of grain size on model performance have tested data ranging from 1m to 10km grain size and found a weak but general decrease in model performance with increasing grain size, although the magnitude and direction of effect appears to be species and area dependent [9, 12, 53–54].

Understanding how well (or not) low resolution models perform with respect to high resolution models has implications for the application of models to spatial management of the marine environment. If high resolution models perform significantly better than low resolution models there may be clear justification for allocation of resources to gather high resolution data such as multibeam bathymetry. The emergence of large-scale high-resolution bathymetry surveys (e.g. the Irish National Seabed Survey, the UKs MAREMAP project, and the Norwegian Mareano project) will provide practitioners with the means to greatly increase model resolution. However, for the vast area of the deep-sea and High Seas, multibeam bathymetry data are unlikely to be available in the near future and lower resolution models may be the only means of highlighting areas where VMEs and associated species are ‘likely to occur’ [9, 15, 55].

#### Comparison of predicted extent

Due to differences in spatial efficiency observed across models of varying environmental data resolution [9, 12] it had been assumed that the area of predicted species distribution would likely increase with the decrease in environmental data resolution (increasing cell size). Our study suggests modelled spatial extent of habitats do vary with data resolution but with no consistent trend in direction nor magnitude of change. This is contrary to previous findings from terrestrial literature where predicted suitable habitat area has repeatedly been shown to increase with increasing cell size [56–60] as a result of the geometric increase of the area of the observed distribution range used to build the HSMs [59]. However, Seo *et al*. [57] and Lauzeral *et al*. [59] demonstrated that this increase in predicted area with increasing cell size depends on species range size and population fragmentation. Therefore we might expect different species to respond differently to an increase in cell size, but to always observe a similar or increased predicted area of distribution with increasing grid cell size as was observed in this study for scleractinian coral and *S*. *fragilissima*. The decreased predicted area of distribution observed for *P*. *carpenteri* at a larger grid cell size is more difficult to explain. Lauzeral *et al*. [59] acknowledge that the effect of grain size on geographic distribution remains to be tested in more detail on real species. This suggests that the use of models by environmental managers for calculating absolute values of extent is ill advised since no generalisation can be made as to the behavior of estimates with increasing data resolution.

#### Comparison of predicted distribution

Comparison of high resolution and low resolution predicted habitat distribution highlighted areas of significant discrepancy between models (Fig 4). While distributions of scleractinian coral in particular did appear to contract around core topographical features in response to an increase in predictor variable resolution this was not the case for *P*. *carpenteri* aggregations or *S*. *fragilissima* aggregations

The distribution of scleractinian coral based on high resolution data produced distinctly different patterns along the continental shelf-edge, along the western slope of Rockall Bank and over the ADS (Fig 4) to that predicted in Ross & Howell [15]. This difference in spatial prediction is likely to be a result of the different emphasis low and high resolution models place on different terrain variables within the final model. For scleractinian coral the most important variable in the low resolution model was bathymetry, while for the high resolution model it was rugosity. The change in cell size may have had a spatial impact on modelled relationships but it also inherently changed the description of a variable and therefore its statistical relationship to the target species. Cold-water coral reef presence has shown strong positive correlation to steep slope values [12, 61] (indeed slope was an important variable to both low and high resolution models), but such values are reduced when derived from low resolution bathymetry, in turn reducing the width of a habitats slope specific niche [9].

Discrepancy between high resolution and low resolution predicted habitat distribution was greatest for *P*. *carpenteri* (Fig 4B). The high resolution model predicted suitable *P*. *carpenteri* environment almost along the entire edge of the continental shelf on the slopes of Rockall Trough and the PSB, which low resolution failed to identify [15] (Fig 4B). Both models predicted the Goban Spur area, south of the PSB as suitable habitat for *P*. *carpenteri*, but the high resolution model predicted a distribution stretching across the entire feature (Fig 4B). The Goban Spur has been observed to support a high number of suspension feeding benthic taxa (1000–1500m depths), predominantly *P*. *carpenteri*, due to high current flow velocities in the area increasing the re-suspension of particulate matter and in turn food availability [24, 62]. It is likely that the high resolution model more closely reflects the known distribution of *P*. *carpenteri*; however without independent validation data it is impossible to know which model provides a more accurate representation of distribution.

A difference in the importance of predictor variables to the final low and high resolution *P*. *carpenteri* models was observed, with slope being important to the higher resolution model. *P*. *carpenteri* occurrence is thought to be influenced by the presence of internal waves related to critical slope angles [23]. It is possible that the high resolution model captured this relationship more fully than the low resolution model but this is speculation.

Environmental data resolution appeared to have the least influence over predicted *S*. *fragilissima* distribution (Fig 4C and 4F). It is possible that those factors that influence the distribution operate at a very broad scale or are equally well reflected at both high and low resolution. The importance of low resolution (750m) variables to the *S*. *fragilissima* model suggests this may be the case (see results and Table A in S3 File).

Both models failed to identify suitable habitat for cold-water coral reef within the SW Rockall NEAFC closure put in place to protect known reef habitat. This suggests that even higher resolution bathymetry still is required to resolve the key influential topographic features (<200m) in this area that are considered important in driving reef distribution (e.g. iceberg plough-marks), and affirms that consideration of the scale of seabed features must be made when making decisions on data resolution in the application of habitat suitability models and MPA assessment [9, 12].

#### Comparison of percentage area protected

It should be noted that for this study full coverage high resolution bathymetry data for the entire MPA network was unavailable (Fig 1). HSM performance has been known to depend on a number of factors as well as bathymetry derived predictor variables such as the inclusion of oceanographic and ocean chemistry models [12], dispersal ranges, and species interactions [56]; a focus for future deep-sea HSM work. Estimates of percentage area protected for the three deep-sea habitats’ are therefore not a complete assessment, but do provide a means to investigate the influence of data resolution for the purpose of assessing conservation management.

Ross & Howell [15] suggest that in using habitat distributions based on low resolution bathymetry data, percentages of predicted suitable environments protected by the MPA network should be taken as maximal figures. However our high resolution models resulted in higher percentage estimates suggesting assessment of percentage area-based conservation targets based on low resolution models result in conservative figures. In consideration of conservation management goals and the progression towards the better protection of deep-sea VMEs, it is better to provide estimates that are overly cautious (based on low resolution bathymetry) in line with the precautionary principle.

This study considers a number of MPAs that were primarily designed for the protection of bedrock ‘reef-like’ assemblages such as *L*. *pertusa* habitat and so is not surprising to see scleractinian coral to be the best protected out of the three habitats. The observed rise in percentage protection levels with the increase in model resolution (Table 3) is difficult to explain but may be related to the fact that, considering the total area of each zone (inside vs outside MPAs), proportionally more of the data used to build the models was situated inside the MPAs than outside, with between 42 and 57% of each of the complete datasets situated inside the MPAs. Our initial thoughts were that this may make model predictions more stable for cells inside vs outside the MPAs. However, this does not appear to be the case when considering the mean and range of standard deviation values (based on repeated model predictions) for cells inside vs outside MPAs. This phenomenon warrants further investigation under simulated conditions. However this is outside the scope of this paper.

The desired application of modelled habitat distributions should be at the forefront of environmental data resolution choice. In the case of politically set percentage targets by which the success, or otherwise, in the protection of habitats (for example the IUCN target of 20–30% representation of each ‘listed’ habitat within strictly protected areas) is measured, a conservative approach to assessment is advantageous. For some habitats high resolution models present a more accurate distribution (as assessed from indices of model performance based on correctly predicting test datasets); such data should be used in the initial stages of conservation management strategy and detailed MPA design at local scales, where low resolution models fail to identify key habitat occurrence. However full coverage high resolution data are largely unavailable and are expensive to obtain. In this case low resolution environmental data should be used in HSM to support higher level decision making at more regional scales, to evaluate progression towards protection targets and to assess the proportion of habitat protected by the conservation management strategies in place.

## Supporting Information

S1 File Raw data sources. (DOC)

S2 File Pre-selection of variables. (DOC)

S3 File Modelling results. (DOC)

## References

[1] Rodríguez JP, Brotons L, Bustamante J, Seoane J. The application of predictive modelling of species distribution to biodiversity conservation. Divers. Distrib. 2007; 13: 243–251.

[2] Guisan A, Zimmermann NE. Predictive habitat distribution models in ecology. Ecol Modell. 2000; 135: 147–186.

[3] Howell KL, Billett DSM, Tyler PA. Depth-related distribution and abundance of sea stars (Echinodermata: Asteroidea) in the Porcupine Seabight and Porcupine Abyssal Plain, N.E. Atlantic. Deep Sea Res Part 1 Oceanogr Res Pap. 2002; 49: 1901–1920.

[4] Howell KL. A benthic classification system to aid in the implementation of marine protected area networks in the deep/high seas of the NE Atlantic. Biol Conserv. 2010; 143: 1041–1056.

[5] Wilson MFJ, O’Connell B, Brown C, Guinan JC, Grehan AJ. Multiscale Terrain Analysis of Multibeam Bathymetry Data for Habitat Mapping on the Continental Slope. Marine Geodesy. 2007; 30: 3–35.

[6] Wolock DM, McCabe GJ. Differences in topographic characteristics computed from 100- and 1000-m resolution digital elevation model data. Hydrol Process. 2000; 14: 987–1002.

[7] Wilson KA, Westphal MI, Possingham HP, Elith J. Sensitivity of conservation planning to different approaches to using predicted species distribution data. Biol Conserv. 2005; 122: 99–112.

[8] Deng Y, Wilson JP, Bauer BO. DEM resolution dependencies of terrain attributes across a landscape. Int J Geogr Inf Sci. 2007; 21: 187–213.

[9] Rengstorf AM, Grehan A, Yesson C, Brown C. Towards High-Resolution Habitat Suitability Modeling of Vulnerable Marine Ecosystems in the Deep-Sea: Resolving Terrain Attribute Dependencies. Marine Geodesy. 2012; 35: 343–361.

[10] Guisan A, Thuiller W. Predicting species distribution: offering more than simple habitat models. Ecol Lett. 2005; 8: 993–1009. 10.1111/j.1461-0248.2005.00792.x 34517687

[11] Davies AJ, Wisshak M, Orr JC, Roberts JM. Predicting suitable habitat for the cold-water coral Lophelia pertusa (Scleractinia). Deep Sea Res Part 1 Oceanogr Res Pap. 2008; 55: 1048–1062.

[12] Rengstorf AM, Yesson C, Brown C, Grehan AJ. High-resolution habitat suitability modelling can improve conservation of vulnerable marine ecosystems in the deep sea. J Biogeogr, 2013; 40: 1702–1714.

[13] De Mol B, Van Rensbergen P, Pillen S, Van Herreweghe K, Van Rooij D, McDonnell A, et al Large deep-water coral banks in the Porcupine Basin, southwest of Ireland. Mar Geol. 2002; 188, 193–231.

[14] Etnoyer P, Morgan LE. Predictive habitat model for deep gorgonians needs better resolution: comment on Bryan & Metaxas (2007). Mar Ecol Prog Ser. 2007; 339: 311–312.

[15] Ross RE, Howell KL. Use of predictive habitat modelling to assess the distribution and extent of the current protection of “listed” deep-sea habitats. Divers. Distrib. 2013; 19: 433–445.

[16] Bryan TL, Metaxas A. Predicting suitable habitat for deep-water gorgonian corals on the Atlantic and Pacific Continental Margins of North America. Mar Ecol Prog Ser, 2007; 330: 113–126.

[17] Marshall C. Species distribution modelling to support marine conservation planning. PhD thesis, University of Plymouth. 2011. Available: http://pearl.plymouth.ac.uk/handle/10026.1/1176

[18] Gallant JC, Dowling TI. A multi-resolution index of valley bottom flatness form mapping depositional areas. Water Resour Res. 2003; 39: 1347–1359.

[19] Rengstorf AM, Mohn C, Brown C, Wisz MS, Grehan AJ. Predicting the distribution of deep-sea vulnerable marine ecosystems using high-resolution data: Considerations and novel approaches. Deep Sea Res Part 1 Oceanogr Res Pap. 2014; 93: 72–82.

[20] Freiwald A, Fosså JH, Grehan A, Koslow T. Roberts JM. Cold-water coral reefs. UNEP-WCMC, Cambridge, UK. 2004. Available: http://www.ourplanet.com/wcmc/pdfs/Cold-waterCoralReefs.pdf

[21] Howell KL, Bullimore RD, Foster NL. Quality assurance in the identification of deep-sea taxa from video and image analysis: response to Henry and Roberts. ICES J Mar Sci. 2014; 4: 899–906.

[22] White M, Mohn C, Stigter H, Mottram G. Deep-water coral development as a function of hydrodynamics and surface productivity around the submarine banks of the Rockall Trough, NE Atlantic In: Freiwald A, Roberts JM, editors. Cold-water corals and ecosystems. Springer, Berlin/Heidelberg; 2005 pp. 503–514.

[23] Rice AL, Thurston MH, New AL. Dense aggregations of a hexactinellid sponge, Pheronema carpenteri, in the Porcupine Seabight (northeast Atlantic Ocean), and possible causes. Prog Oceanogr. 1990; 24: 179–196.

[24] Hughes DJ, Gage JD. Benthic metazoan biomass, community structure and bioturbation at three contrasting deep-water sites on the northwest European continental margin. Prog Oceanogr. 2004; 63: 29–55.

[25] White M. Comparison of near seabed currents at two locations in the Porcupine Sea Bight–implications for benthic fauna. J Mar Biol Assoc U.K. 2003; 8: 683–686.

[26] Bett BJ. UK Atlantic Margin Environmental Survey: Introduction and overview of bathyal benthic ecology. Cont Shelf Res. 2001; 21: 917–956.

[27] Hughes JA, Gooday AJ. Associations between living benthic foraminifera and dead tests of Syringammina fragilissima (Xenophyophorea) in the Darwin Mounds region (NE Atlantic). Deep Sea Res Part 1 Oceanogr Res Pap. 2004; 51: 1741–1758.

[28] ESRI. ArcGIS, version 9.3.1. ESRI, Redlands, CA. 2009.

[29] Wright DJ, Lundblad ER, Larkin EM, Rinehart RW, Murphy J, Cary-Kothera L, Draganov K. ArcGIS Benthic Terrain Modeler Oregon State University, Davey Jones Locker Seafloor Mapping/Marine GIS Laboratory and NOAA Coastal Services Center, Corvallis, OR 2005.

[30] Kostylev VE, Erlandsson J, Ming MY, Williams GA. The relative importance of habitat complexity and surface area in assessing biodiversity: Fractal application on rocky shores. Ecological Complexity. 2005; 2: 272–286.

[31] Guinan J, Brown C, Dolan MFJ, Grehan AJ. Ecological niche modelling of the distribution of cold-water coral habitat using underwater remote sensing data. Ecol Inform. 2009; 4: 83–92.

[32] Lahoz-Monfort JJ, Guillera-Arroita G, Wintle BA. Imperfect detection impacts the performance of species distribution models. Global Ecol Biogeogr. 2014; 23: 504–515.

[33] Guillera-Arroita G, Lahoz-Monfort JJ, Elith J, Gordon A, Kujala H, Lentini PE, et al Is my species distribution model fit for purpose? Matching data and models to applications. Global Ecol Biogeogr. 2015; 24: 276–292.

[34] Phillips SJ, Anderson RP, Schapire RE. Maximum entropy modelling of species geographic distributions. Ecol Modell. 2006; 190: 231–259.

[35] Elith J, Graham CH, Anderson RP, Miroslav D, Ferrier S, Guisan A, et al Novel methods improve prediction of species’ distributions from occurrence data. Ecography. 2006; 29: 129–151.

[36] Roberts JJ, Best BD, Dunn DC, Treml EA, Halpin PN. Marine Geospatial Ecology Tools: an integrated framework for ecological geoprocessing with ArcGIS, Python, R, MATLAB, and C++. Environ Modell & Softw. 2010; 25: 1197–1207.

[37] Phillips SJ, Dudik M. Modelling of species distributions with MaxEnt : new extensions and a comprehensive evaluation. Ecography. 2008; 31: 161–175.

[38] Howell KL, Holt R, Endrino IP, Stewart H. When the species is also a habitat: comparing the predictively modelled distributions of Lophelia pertusa and the reef habitat it forms. Biological Conservation. 2011; 144: 2656–2665.

[39] Hijmans RJ, Phillips S, Leathwick J, Elith J. Dismo: Species Distribution Modeling. R Package Version 0.6–10. 2011. Available at: http://CRAN.R-project.org/package=dismo.

[40] Freeman E. PresenceAbsence: an R package for Presence–Absence model evaluation. USDA Forest Service, Rocky Mountain Research Station. 2007. Available at: http://CRAN.R-project.org/package=PresenceAbsence.

[41] R Development Core Team. R: A language and environment for statistical computing R Foundation for Statistical Computing, Vienna, Austria 2013 Available at: http://www.R-project.org/

[42] Lobo JM, Jiménez-Valverde A, Real R. AUC: a misleading measure of the performance of predictive distribution models. Glob. Ecol. Biogeogr. 2008; 17: 145–151.

[43] Peterson AT, Papes M, Soberón J. Re-thinking receiver operating characteristic analysis applications in ecological niche modelling. Ecol Modell. 2008; 213: 63–72.

[44] Jiménez-Valverde A. Insights into the area under the receiver operating characteristic curve (AUC) as a discrimination measure in species distribution modelling. Glob. Ecol. Biogeogr. 2012; 21: 498–507.

[45] Fielding AH, Bell JF. A review of methods for the assessment of prediction errors in conservation presence/absence models. Environmental Conservation. 1997; 24: 38–49.

[46] Liu C, Berry PM, Dawson TP, Pearson RG. Selecting thresholds of occurrence in the prediction of species distributions. Ecography. 2005; 28: 385–393.

[47] Cantor SB, Sun CC, Tortolero-Luna G, Richards-Kortum R, Follen M. A comparison of C/B ratios from studies using receiver operating characteristics curve analysis. J Clin Epidemiol. 1999; 52: 885–892. 10529029 10.1016/s0895-4356(99)00075-x

[48] Manel S, Williams HC, Ormerod SJ. Evaluating presence-absence models in ecology: the need to account for prevalence. J App Ecol. 2001; 38: 921–931.

[49] Manel S, Dias JM, Ormerod SJ. Comparing discriminant analysis, neural networks and logistic regression for predicting species distributions: a case study with a Himalayan river bird. Ecol Modell. 1999; 120: 337–347.

[50] Ferrier S, Watson G. An evaluation of the effectiveness of environmental surrogates and modelling techniques in predicting the distribution of biological diversity. Consultancy report to the Biodiversity Convention and Strategy Section of the Biodiversity Group, Environment Australia. Environment Australia, Arimidale. 1997.

[51] Tobaiske C. Effects of spatial scale on the predictive ability of habitat models for the Green Woodpecker in Switzerland In: Scott JM, Heglund PJ, Morrison ML, editors. Predicting Species Occurences; Issues of accuracy and scale. Island Press, Washington, D.C. 2002 pp. 63–72.

[52] Graf RF, Bollmann K, Sachot S, Suter W, Bugmann H. On the generality of habitat distribution models: a case study of capercaillie in three Swiss regions. Ecography. 2006; 29: 319–328.

[53] Guisan A, Zimmermann NE, Elith J, Graham CH, Phillips S, Peterson AT. What matters for predicting the occurrences of trees: Techniques, data, or species' characteristics? Ecol Monogr. 2007; 77: 615–630.

[54] Gottschalk TK, Aue B, Hotes S, Ekschmitt K. Influence of grain size on species–habitat models. Ecol Modell. 2011; 222: 3403–3412.

[55] Yesson C, Taylor ML, Tittensor DP, Davies AJ, Guinotte J, Baco A, et al Global habitat suitability of cold-water octocorals. J Biogeogr. 2012; 39: 1278–1292.

[56] McPherson JM, Jetz W. Effects of species’ ecology on the accuracy of distribution models. Ecography. 2007; 30: 135–151.

[57] Seo C, Thorne JH, Hannah L, Thuiller W. Scale effects in species distribution models: implication for conservation planning under climate change. Biol Lett. 2009; 5: 39–43. doi: 10.1098/rsbl.2008.0476 18986960 10.1098/rsbl.2008.0476 PMC2657743

[58] Hu J, Jiang Z. Predicting the potential distribution of the endangered Przewalski’s gazelle. J Zool. 2010; 282: 54–63.

[59] Lauzeral C, Grenouillet G, Brosse S. Spatial range shape drives the grain size effects in species distribution models. Ecography. 2013; 36: 778–787.

[60] Song W, Kim E, Lee D, Lee M, Jeon S-W. The sensitivity of species distribution modelling to scale differences. Ecol Modell. 2013; 248: 113–118.

[61] Guinan JC, Grehan AJ, Dolan MFJ, Brown C. Quantifying relationships between video observations of cold-water coral and seafloor features in Rockall Trough, west of Ireland. Mar Ecol Prog Ser. 2009; 375: 125–138.

[62] Flach E, Lavaleye M, Stigter H, Thomsen L. Feeding types of the benthic community and particle transport across the slope of the N.W. European continental margin (Goban Spur). Prog Oceanogr. 1998; 42: 209–231.

