# Supplementary material for: The Influence of Data Resolution on Predicted Distribution and Estimates of Extent of Current Protection of Three ‘Listed’ Deep-Sea Habitats
Source: PLoS One. 2015 Oct 23;10(10):e0140061. doi: 10.1371/journal.pone.0140061 (PMC4619891; doi:10.1371/journal.pone.0140061)
Supplement: S1 File — (DOC) [file pone.0140061.s001.doc]

**SUPPORTING INFORMATION**

**The influence of data resolution on predicted distribution and estimates of extent of current protection of three ‘listed’ deep-sea habitats**

Lauren K. Ross, Rebecca E. Ross, Heather A. Stewart and Kerry L. Howell

**S1 File - Raw data sources**

**Text A.** Data sources for presence / pseudo absence datasets (1-14).

**Table A. Sources and details of high resolution multi-beam bathymetry data.**

| Data set | Reference | Location | Native cell size | Projection of source file |
| --- | --- | --- | --- | --- |
| BAS | [15] | Rosemary Bank | 100m | ED 1950 UTM (29N) |
| CD118 UNCLOS | [16] | Hatton | 100m | WGS84 UTM (27N) |
| CD174 | [17] | Rockall Trough surrounding Anton Dohrn Seamount | 0.001° | WGS84 |
| CD91 LOIS | [18] | Barra Fan | 0.0025° | WGS84 |
| SEA-SAC2006 | [19, 20] | Rosemary Bank, Hatton Bank, George Bligh Bank, Wyville Thomson Ridge | 25m | UTM (27N, 28N, 29N, 30N) |
| IFREMER | Not available | Hebrides Terrace/Donegal Fan | 0.0025° | WGS84 |
| MESH | [21, 22] | SW Approaches, Explorer and Dangaard Canyons | 25m | UTM (29N) |
| NISS | Not available | South Hebrides Terrace to Rockall Bank | 0.002° | WGS84 |
| NISS | Not available | Rockall Bank approx. Haddock Box | 0.000225° | WGS84 |
| SEA7_KJ2005 | [23] | Anton Dohrn Seamount, Rockall Bank, George Bligh Bank, Hatton Bank, Hatton-Rockall Basin, Rockall Trough, Rosemary Bank | 0.0002° | WGS84 |
| Irish National Seabed Survey | <http://www.infomar.ie/> | Whole Irish deep-water area | 111m | WGS84 |

**S1 File References**

1. Rice AL. RRS Discovery Cruise 185, 18 August–17 September 1989. Abyssal benthic biology at the European Community Station (48,50′N 16,30′W). Institute of Oceanographic Sciences Deacon Laboratory, Wormley, UK. 1990.
2. Rice AL, Thurston MH, New AL. Dense aggregations of a hexactinellid sponge, Pheronema carpenteri, in the Porcupine Seabight (northeast Atlantic Ocean), and possible causes. Progress in Oceanography. 1990; 24: 179-196.
3. Jackson PAB, Thurston MH, Rice AL. Station data from the IOS Benthic Biological Survey of the Porcupine Seabight region (NE Atlantic) 1977–89. Institute of Oceanographic Sciences Deacon Laboratory, Wormley, UK. 1991.
4. Rice AL. RRS Challenger Cruise 79, 12 May–03 June 1991. Benthic biology at the European Community Station (48,50′N 16,30′W) and in the Porcupine Seabight. Institute of Oceanographic Sciences Deacon Laboratory, Wormley, UK. 1992.
5. Bett BJ. RRS Challenger Cruise 111, 29 March-25 April 1994. Benthic biology of the Porcupine Abyssal Plain (48,50′N 16,30′W). Institute of Oceanographic Sciences Deacon Laboratory, Wormley, UK. 1994.
6. Billett DSM. RRS Challenger Cruise 142 19 Apr-19 May 1999. Temporal and spatial variability of benthic communities on the Porcupine Abyssal Plain and in the Porcupine Seabight. Southampton Oceanography Centre, Southampton, UK. 2000.
7. Bett BJ, Billett DSM, Masson DG, Tyler PA. RRS Discovery Cruise 248 07 Jul–10 Aug 2000. A multidisciplinary study of the environment and ecology of deep-water coral ecosystems and associated seabed facies and features (The Darwin Mounds, Porcupine Bank and Porcupine Seabight). Southampton Oceanography Centre, Southampton, UK. 2001.
8. Narayanaswamy BE, Howell KL, Hughes DJ, Davies JS, Roberts JM, Black KD. Strategic environmental assessment area 7 photographic analysis report. Department of Trade and Industry, London, UK. NEAFC. 2006.
9. Howell KL, Davies JS, Hughes DJ, Narayanaswamy BE. Strategic environmental assessment/special area for conservation photographic analysis report. Department of Trade and Industry, London, UK. 2007.
10. Howell KL, Davies JS, Narayanaswamy BE. Identifying deep-sea megafaunal epibenthic assemblages for use in habitat mapping and marine protected area network design. Journal of the Marine Biological Association of the United Kingdom. 2009; 90: 33–68.
11. Howell KL, Davies JS, Jacobs C, Narayanaswamy BE. Broadscale survey of the habitats of Rockall Bank, and mapping of Annex I “Reef” habitat. Joint Nature and Conservation Committee, Peterborough, UK. 2009.
12. Stewart H, Davies JS, Long D, Strömberg H, Hitchen K. JNCC Offshore Natura Survey: Anton Dohrn Seamount and East Rockall Bank. Joint Nature and Conservation Committee, Peterborough, UK. 2009.
13. Howell KL, Holt R, Endrino IP, Stewart H. When the species is also a habitat: comparing the predictively modelled distributions of Lophelia pertusa and the reef habitat it forms. Biological Conservation. 2011; 144: 2656–2665.
14. Huvenne VAI. RRS James Cook Cruise 60, 09 May– 12 June 2011, Benthic habitats and the impact of human activities in Rockall Trough, on Rockall Bank and Hatton Basin. National Oceanography Centre Southampton, Southampton, UK. 2011.
15. Howe JA, Stoker MS, Masson DG, Pudsey CJ, Morris P, Larter RD, Bulat J. Seabed morphology and the bottom-current pathways around Rosemary Bank seamount, northern Rockall Trough, North Atlantic. Marine and Petroleum Geology. 2006; 23: 165-181.
16. MacLachlan SE, Elliot GM, Parson LM. Investigations of the bottom current sculpted margin of Hatton bank, NE Atlantic. Marine Geology. 2008; 253:170–184.
17. Wallis DG. Rockall-North Channel MESH geophysical survey, RRS Charles Darwin Cruise CD174, BGS Project 05/05 Operations Report. 2005.
18. McCartney BS, Huthnance JM. RRS Charles Darwin Cruise CD91, 2 March–2 April1995 LOIS Shelf Edge Study. Proudman Oceanogr. Lab. Cruise Rep, 20. 1995.
19. Jacobs CL, Howell KL. MV Franklin Cruise 0206, 03-23 Aug 2006. Habitat investigations within the SEA4 and SEA7 areas of the UK continental shelf. Southampton, UK, National Oceanography Centre Southampton, 95pp. (National Oceanography Centre Southampton Research and Consultancy Report 24). 2007.
20. Stewart, HA, Davies JS. Habitat investigations within the SEA7 and SEA4 areas of the UK continental shelf (Hatton Bank, Rosemary Bank, Wyville Thomson Ridge and Faroe–Shetland Channel). British Geological Survey Commissioned Report, CR/07/051. 85pp. 2007.
21. Stewart HA, Davies JS. SW Approaches MESH Survey, R/V Celtic Explorer Cruise CE0705, BGS Project 07/06, Operations Report. British Geological Survey Commercial Report CR/07/123. 2007.
22. Davies J, Guinan J, Howell K, Stewart H, Verling E. MESH South West Approaches Canyons Survey (MESH Cruise 01-07-01) Final Report. Mapping European Seabed Habitats (MESH) Project Report. 156p. 2008.
23. Jacobs CL. SV Kommandor Jack Cruise 01/05, 11 Jul – 08 Aug 2005. Multibeam bathymetry and high resolution sidescan sonar surveys within the SEA7 area of the UK continental shelf. Southampton, UK, National Oceanography Centre Southampton, 50pp. (National Oceanography Centre Southampton Research and Consultancy Report 7). 2005.
