# Supplementary material for: The Influence of Data Resolution on Predicted Distribution and Estimates of Extent of Current Protection of Three ‘Listed’ Deep-Sea Habitats
Source: PLoS One. 2015 Oct 23;10(10):e0140061. doi: 10.1371/journal.pone.0140061 (PMC4619891; doi:10.1371/journal.pone.0140061)
Supplement: S2 File — (DOC) [file pone.0140061.s002.doc]

**SUPPORTING INFORMATION**

**The influence of data resolution on predicted distribution and estimates of extent of current protection of three ‘listed’ deep-sea habitats**

Lauren K. Ross, Rebecca E. Ross, Heather A. Stewart and Kerry L. Howell

**S2 File - Pre-selection of variables**

**Text A. Variable Pre-selection Methods**

A Pearson product-moment correlation coefficient test was run using the statistical software environment R, version 2.13.1 [1] in order to identify correlated variables. Correlation coefficients >=0.7 were identified as ‘highly correlated’ (Table A).

In order to select the best combination of variables to produce the strongest models, the relationships between individual variables and habitat occurrence were explored and checked for their significance using Binomial Generalised Additive Models (GAMs). GAMs were run with the logit link function in R using the MGCV library [2], employing 4 knots and a gamma of 1.4 to reduce over fitting as advocated by Kim & Gu [3]. Variables showing no significant relationship were excluded from the final MaxEnt model. Individual GAM plots (Figure A) were studied and relationships were considered for biological relevance and reliability in terms of 95% confidence intervals.

The dredge function within the MuMIn library in R [4] was used to explore a variety of global GAMs containing only one of each correlate set in order to confirm correct exclusion of correlated variables. The model containing the most variables within 2 corrected Aikike’s Information Criterion, or AICc, (delta<2) of the top ranked model was employed as the variable combination used in the final MaxEnt model.

**Table A. Highly correlated variable pairs and their correlation coefficients.**

|  |  | Pearson correlation coefficient | | |
| --- | --- | --- | --- | --- |
| Variable | Correlate | a) | b) | c) |
| Bathymetry (200m) | Bathymetry (750m) | 0.99 | 0.99 | 0.99 |
| BPI broad (200m) | BPI broad (750m) | 0.79 | 0.78 | 0.79 |
| Curvature (200m) | Profile curvature (200m) | -0.89 | -0.89 | -0.89 |
| Rugosity (200m) | Slope (200m) | 0.96 | 0.96 | 0.97 |
| Rugosity (200m) | Rugosity (750) | 0.76 | 0.77 | 0.78 |
| Rugosity (200m) | Slope (750m) | 0.72 | 0.73 | 0.75 |
| Slope (200m) | Rugosity (750) | 0.78 | 0.78 | 0.78 |
| Slope (200m) | Slope (750) | 0.74 | 0.75 | 0.75 |
| Curvature (750m) | Profile curvature (750m) | -0.99 | -0.99 | -0.99 |
| Rugosity (750m) | Slope (750) | 0.98 | 0.99 | 0.99 |

a) scleractinian cold-water coral reef; b) *Pheronema carpenteri* aggregations; c) *Syringammina fragilissima* aggregations.

**Figure A. Partial-residual plots of individual variable generalised additive models (GAMs).** GAMs built with individual environmental variables (at both 200m and 750m resolution) against the presence and absence of a) scleractinian cold-water coral reef, b) *Pheronema carpenteri* aggregations, and c) *Syringammina fragilissima* aggregations. Plots illustrate the capacity of each individual variable to predict habitat occurrence which is expressed by deviance away from the no relationship zero line; deviance above zero is a predicted presence and deviance below zero is a predicted absence. Dotted lines are 95% confidence intervals and rugs along the x axis represent the spread of data of which the models are built. (bathy_200 = bathymetry 200m; bathy_750 = bathymetry 750m; bsbpi_200 = broad scale bathymetric position index 200m; bsbpi_750 = broad scale bathymetric position index 750m; curv_200 = curvature 200m; curv_750 = curvature 750m; fsbpi_200 = fine scale bathymetric position index 200m; fsbpi_750 = fine scale bathymetric position index 750m; planc_200 = plan curvature 200m; planc_750 = plan curvature 750m; profc_200 = profile curvature 200m; profc_750 = profile curvature 750m; rug_200 = rugosity 200m; rug_750 = rugosity 750m; slope_200 = slope 200m; slope_750 = slope 750m).

**a)**

**
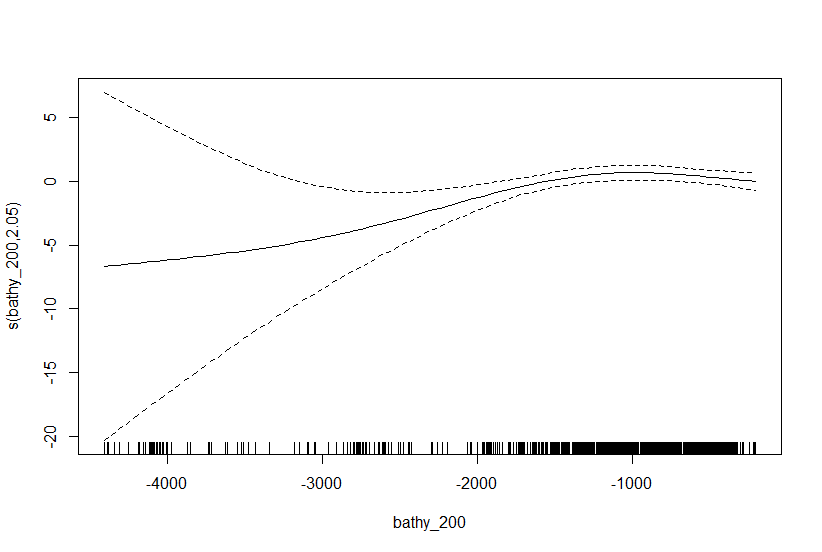
**

**
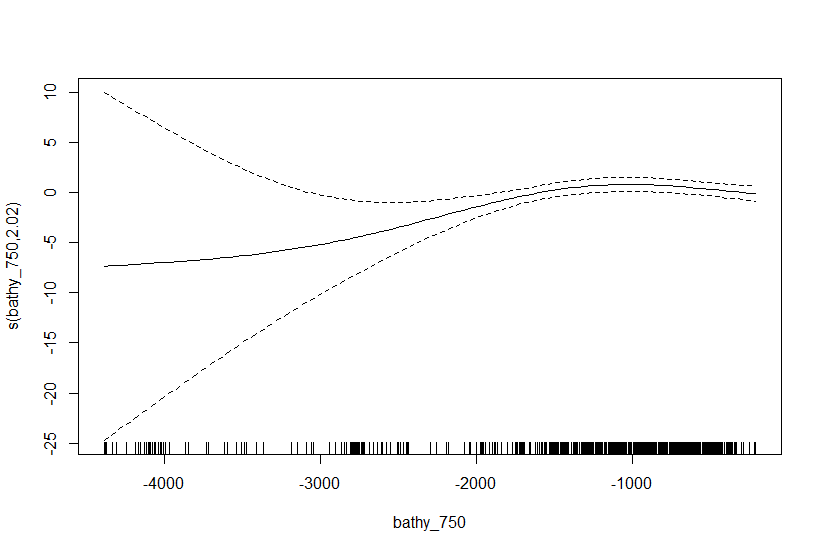
**

**
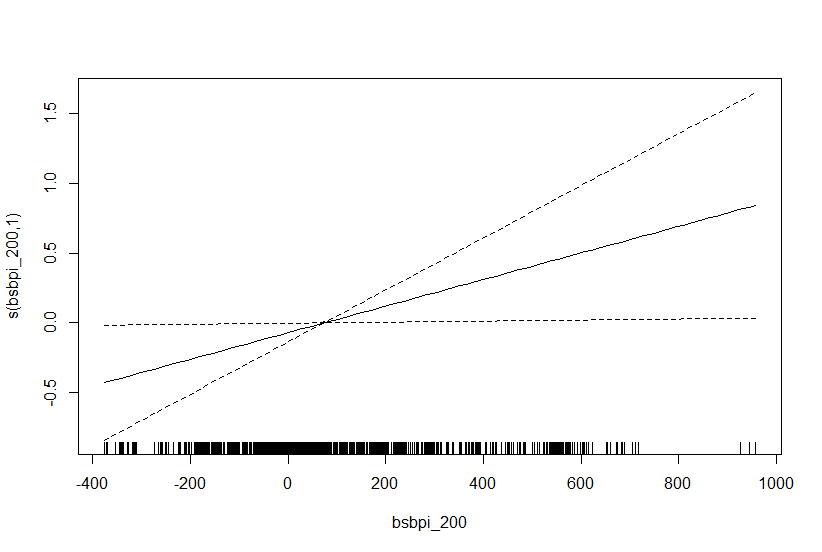
**

**
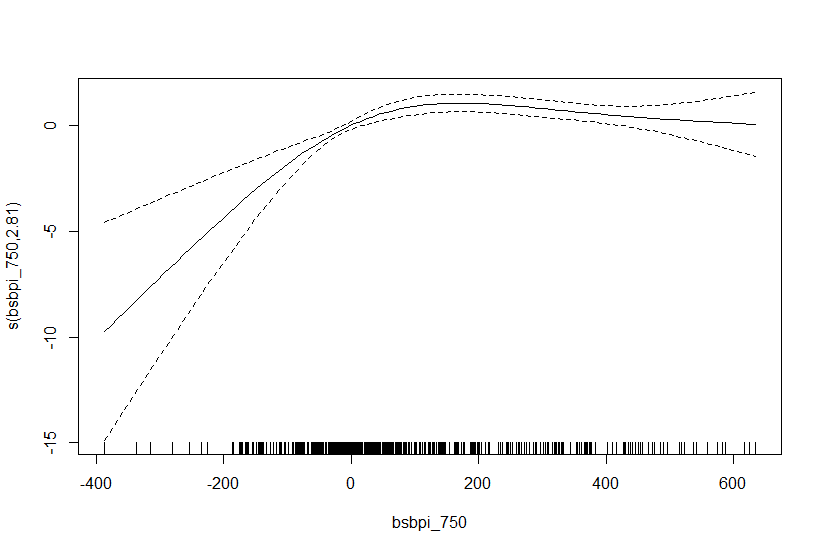
**

**
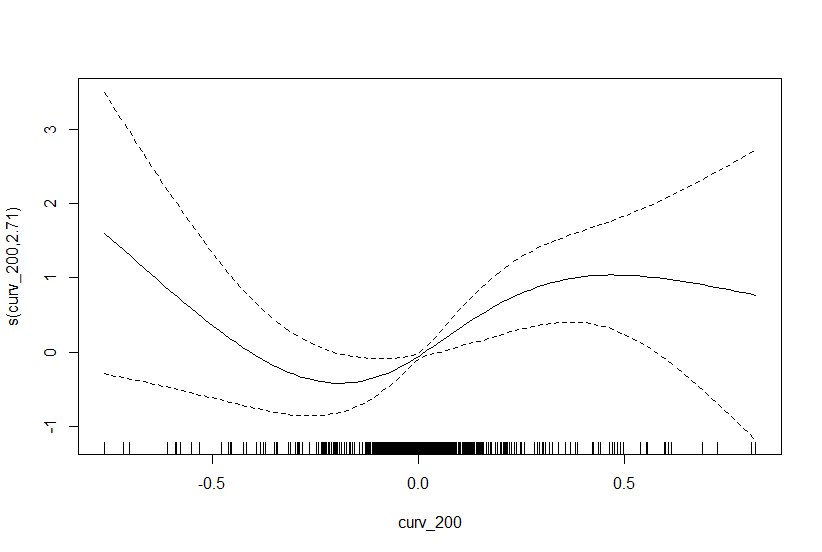
**

**
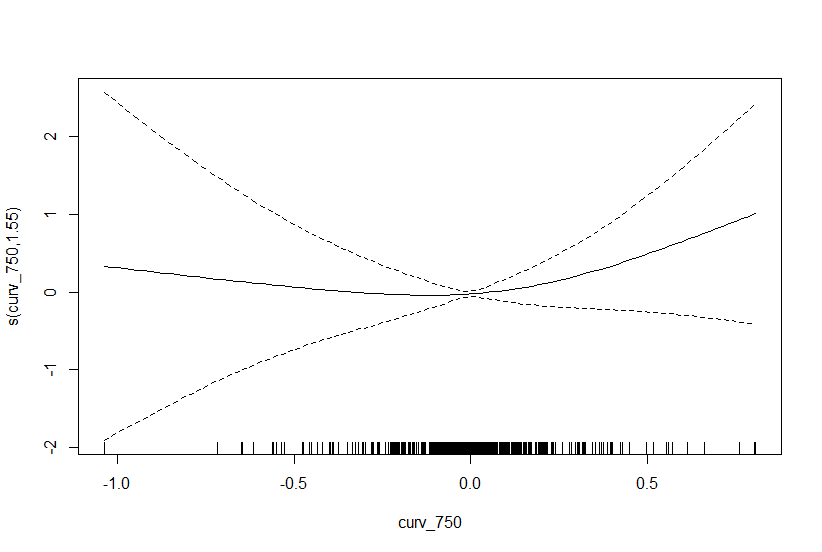
**

**
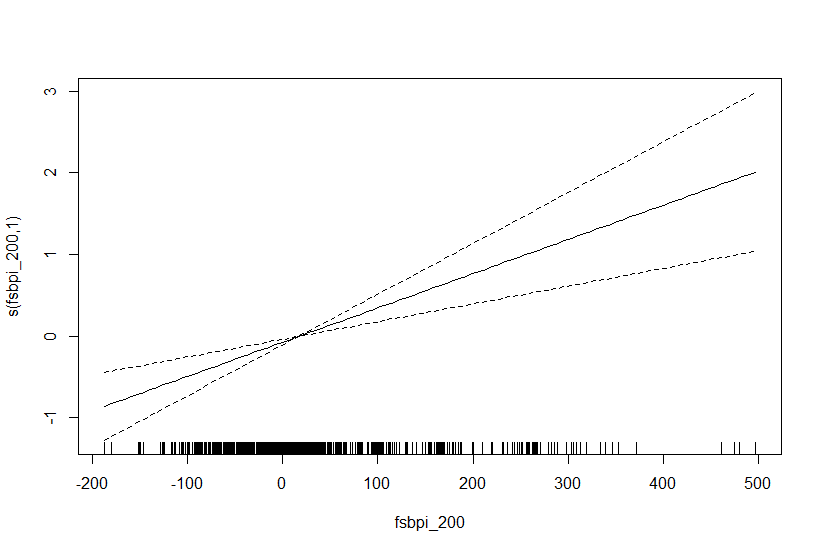
**

**
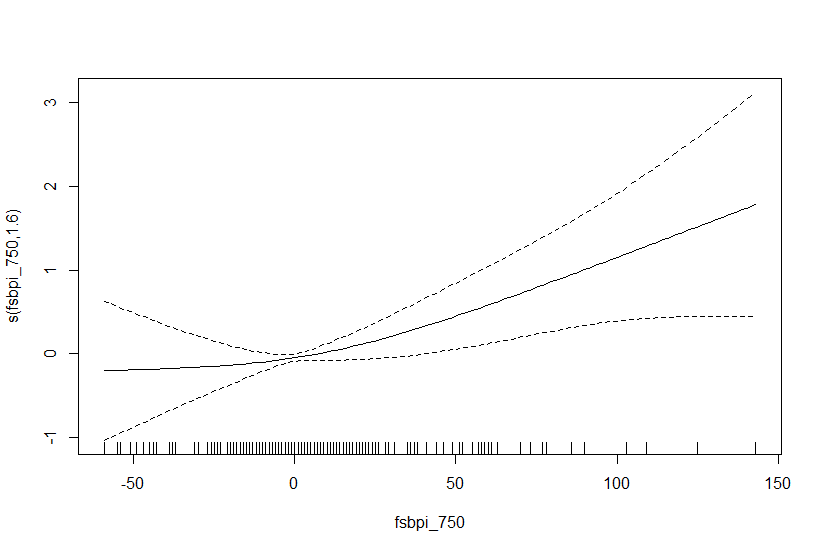
**

**
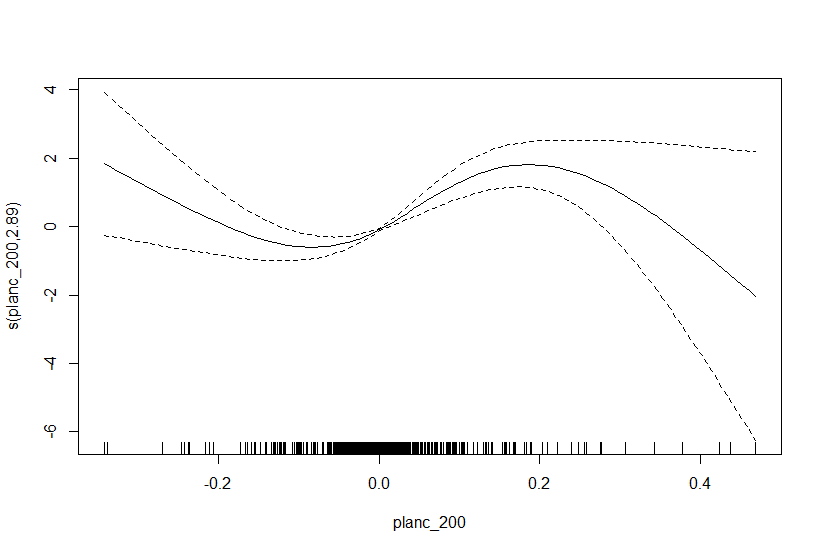
**

**
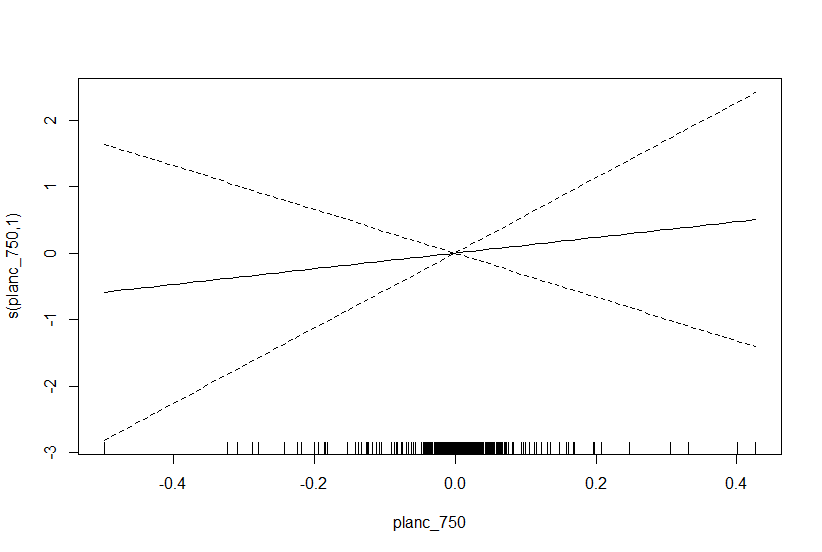
**

**
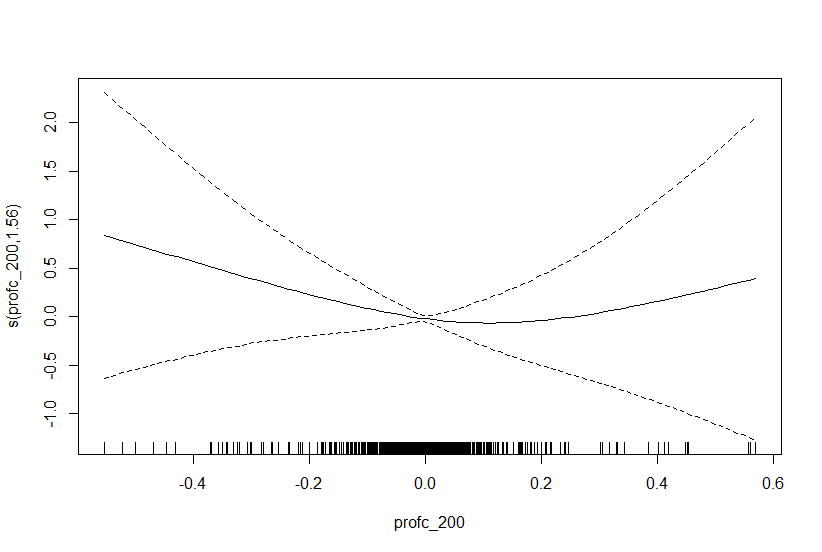
**

**
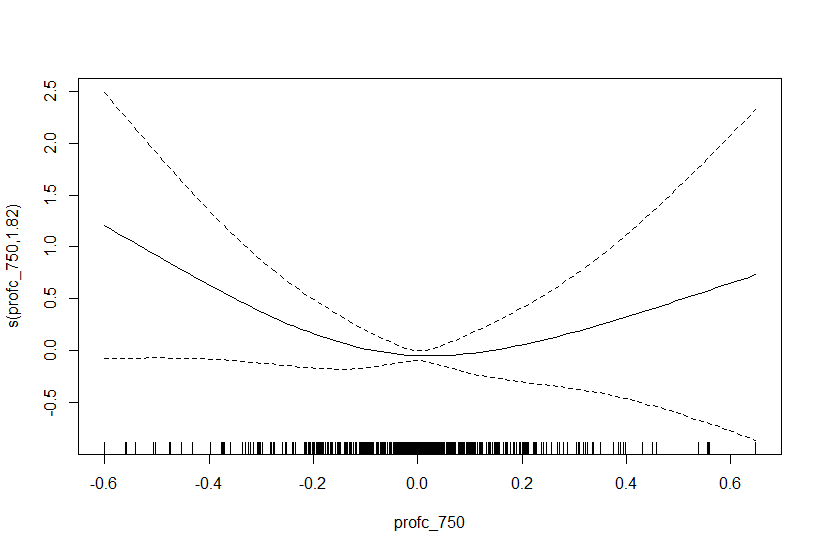
**

**
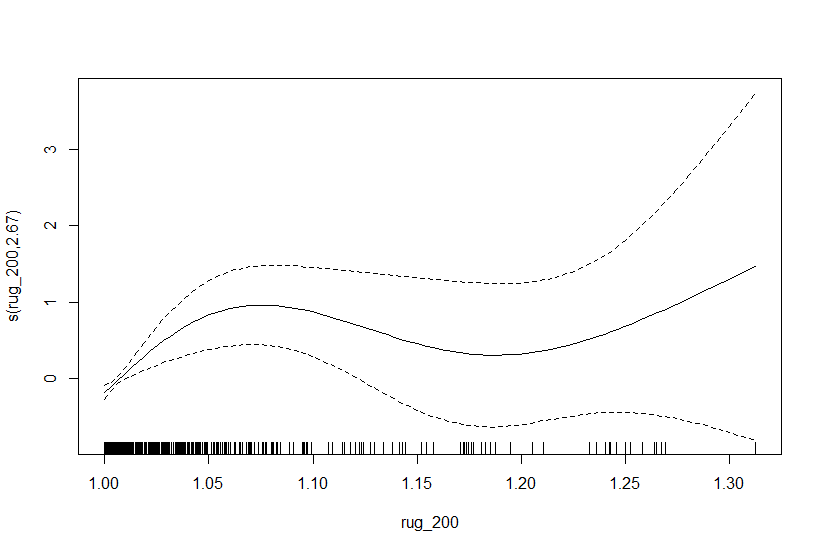
**

**
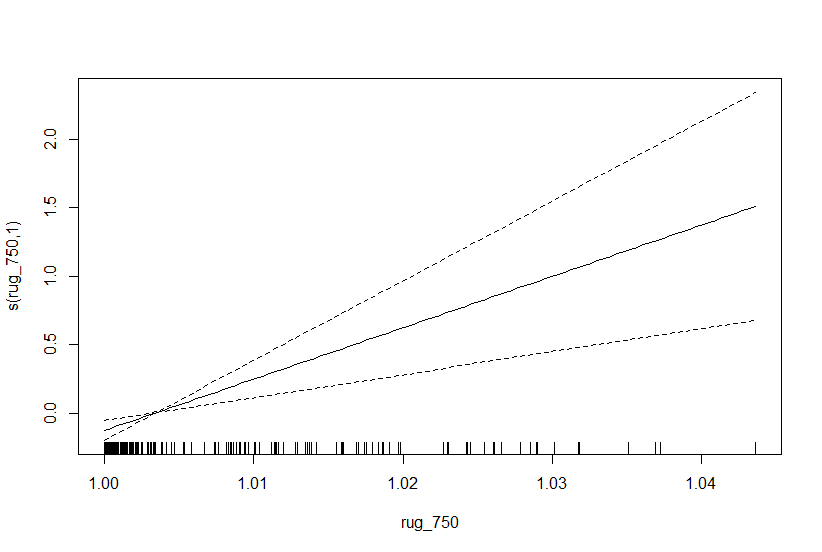
**

**
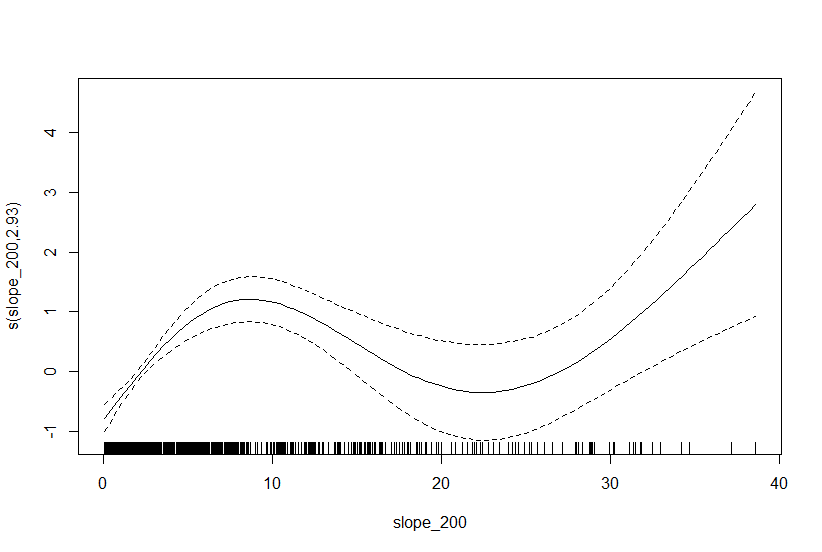
**

**
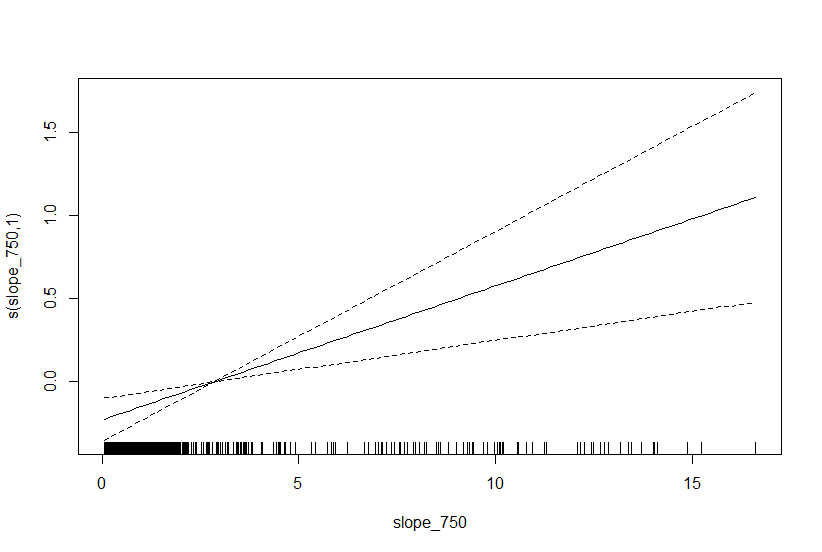
**

**b)**

**
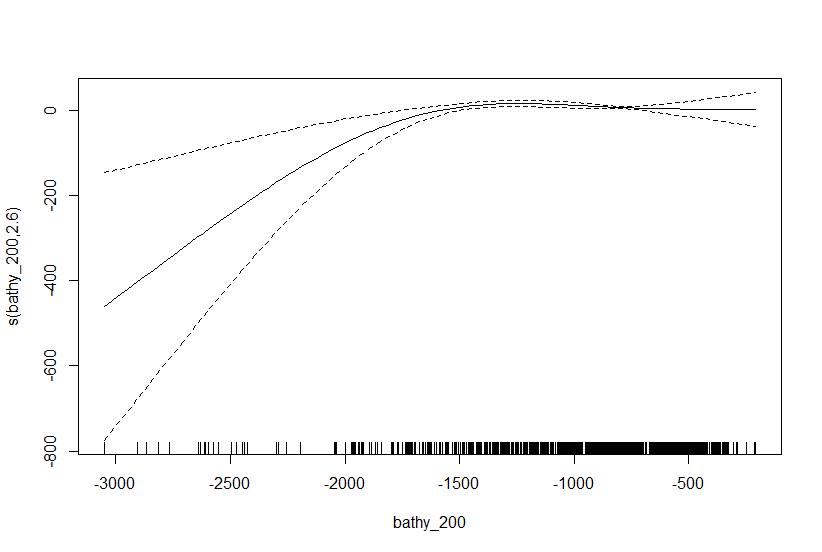
**

**
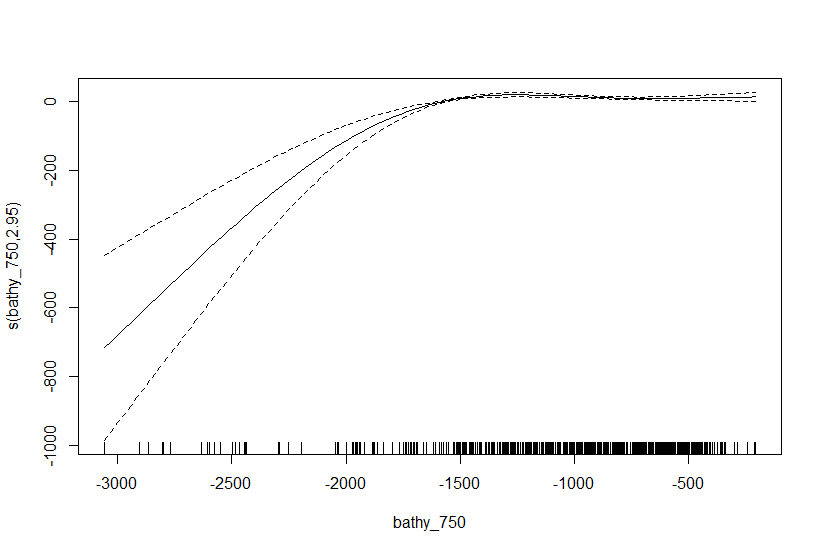
**

**
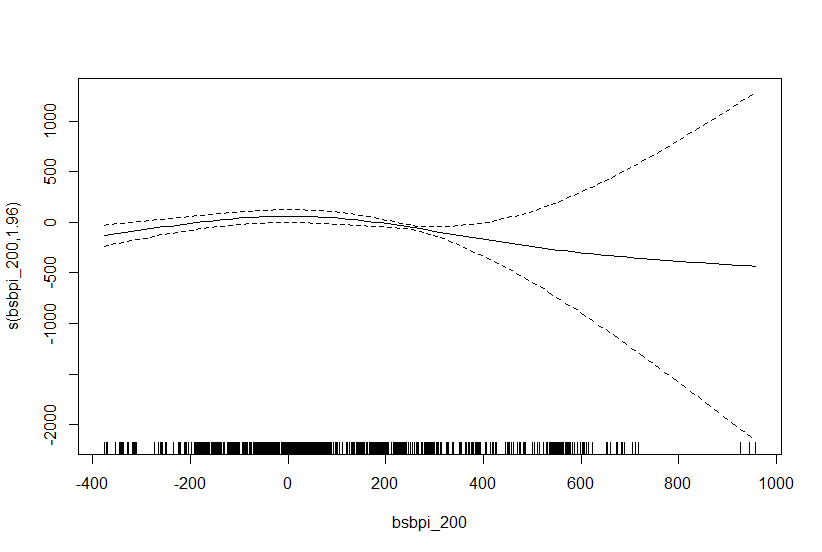
**

**
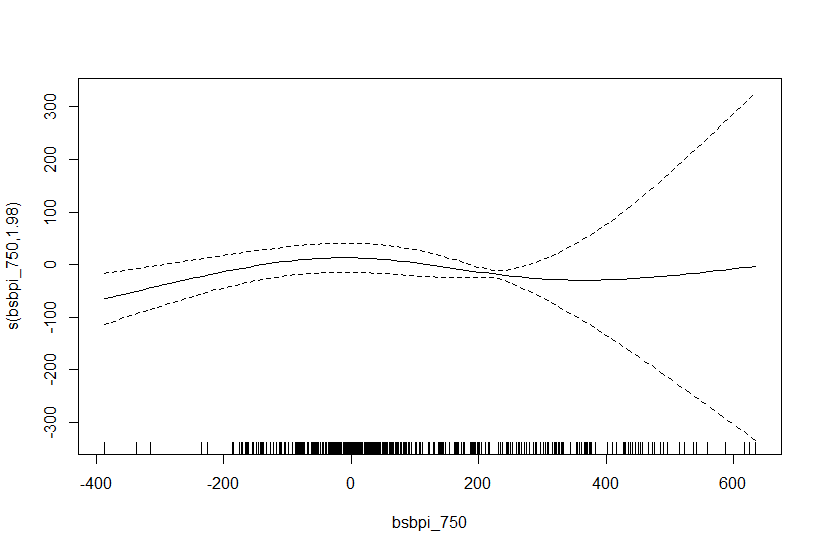
**

**
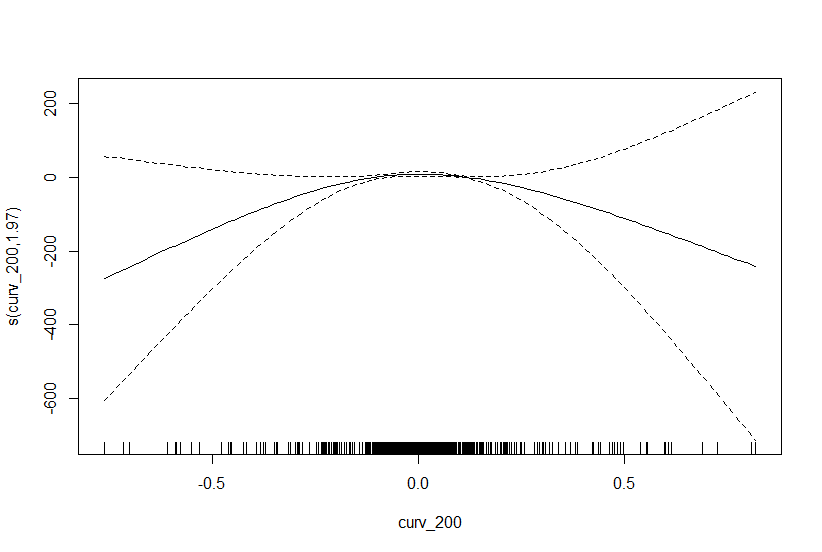
**

**
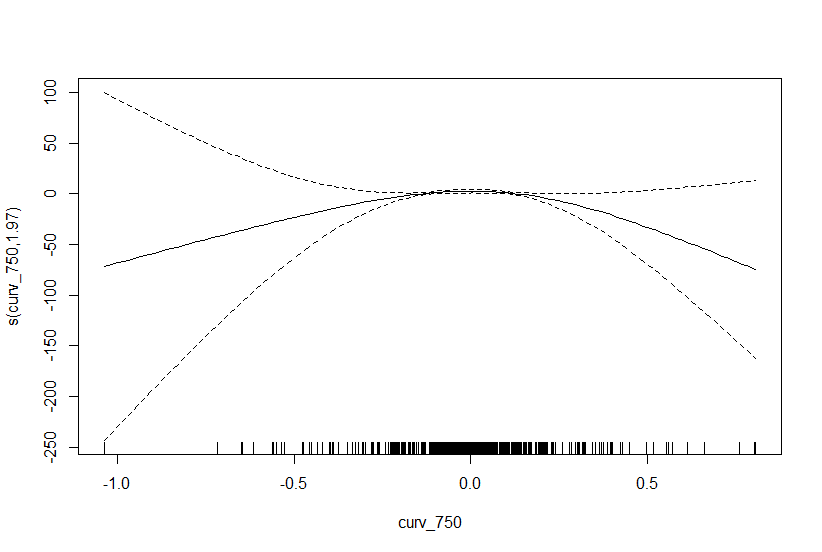
**

**
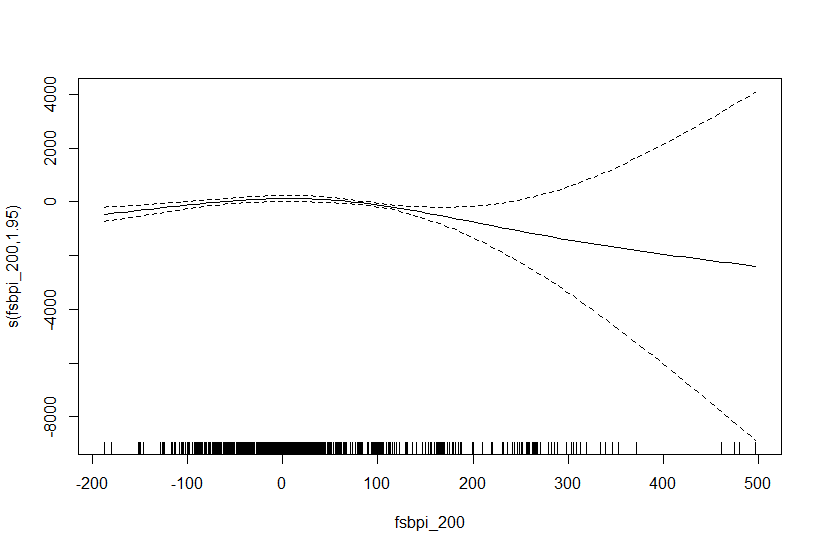
**

**
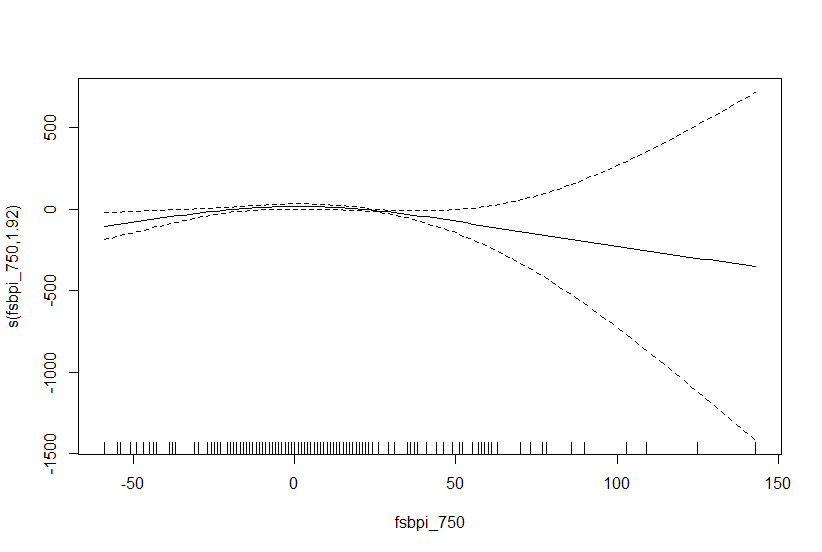
**

**
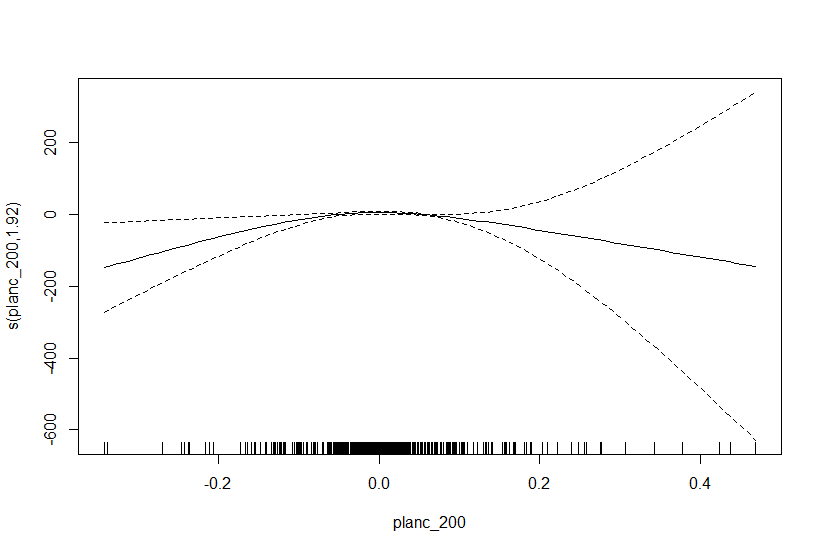
**

**
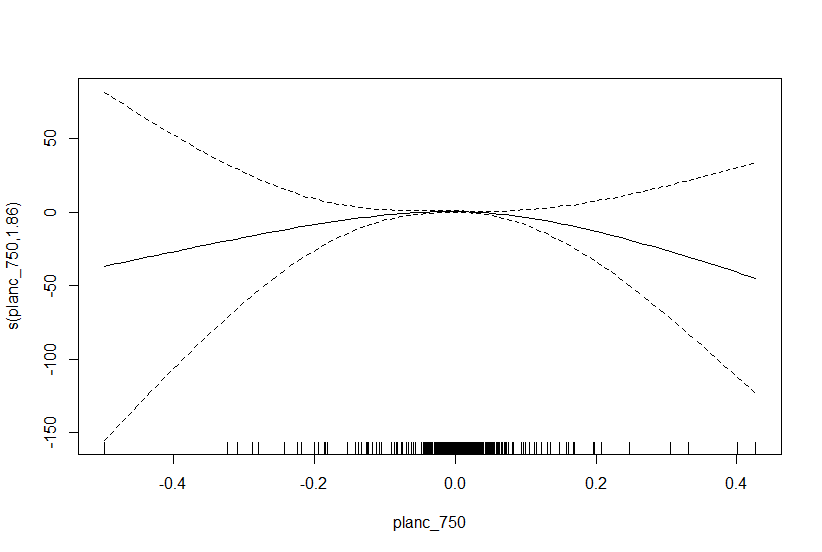
**

**
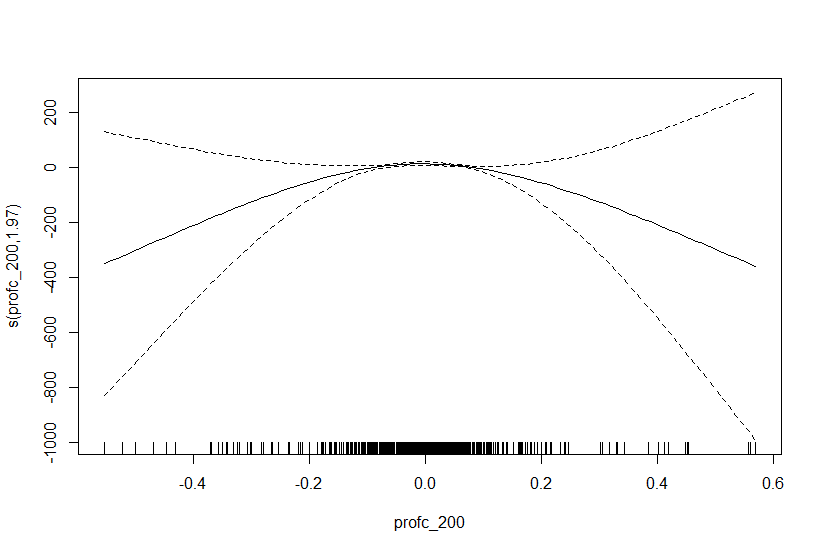
**

**
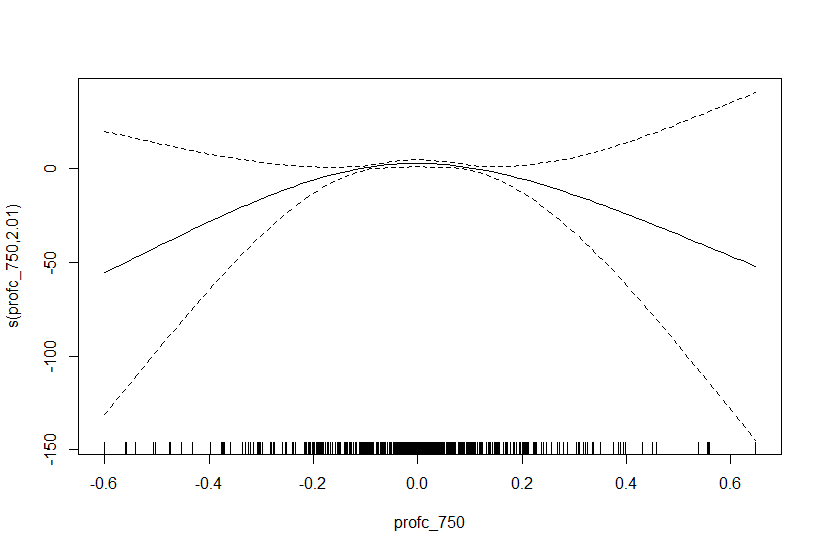
**

**
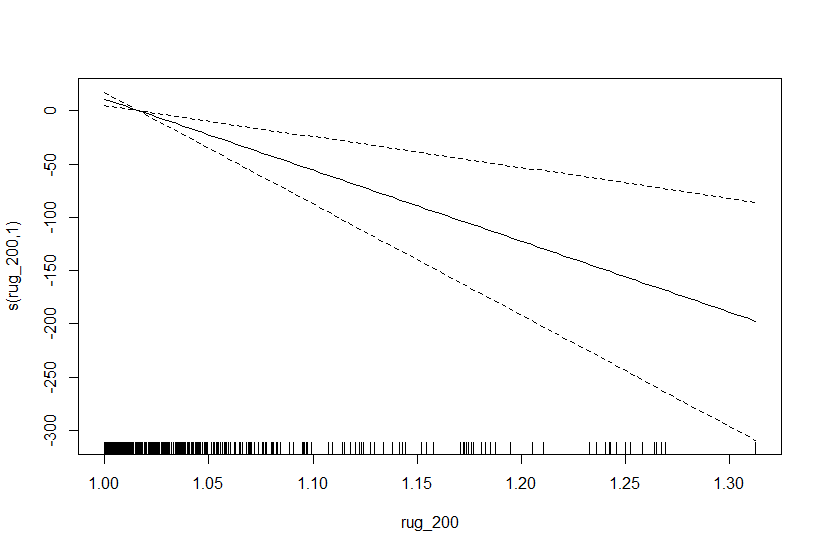
**

**
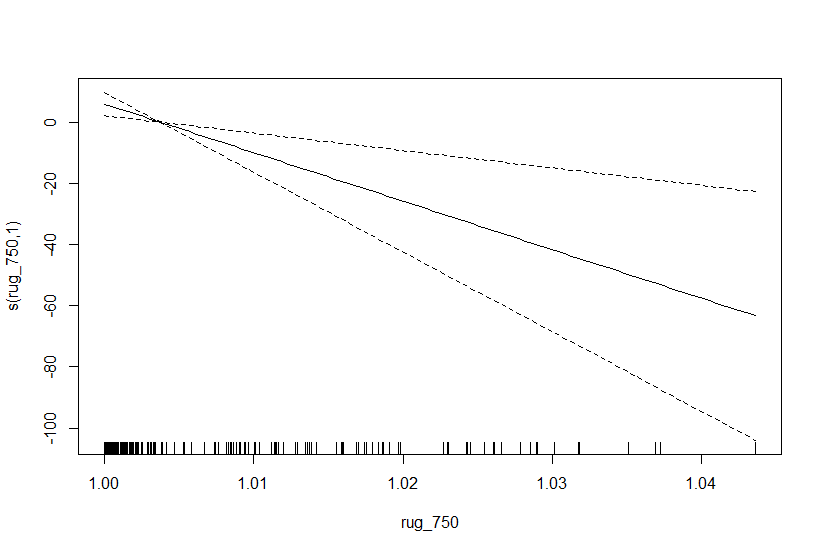
**

**
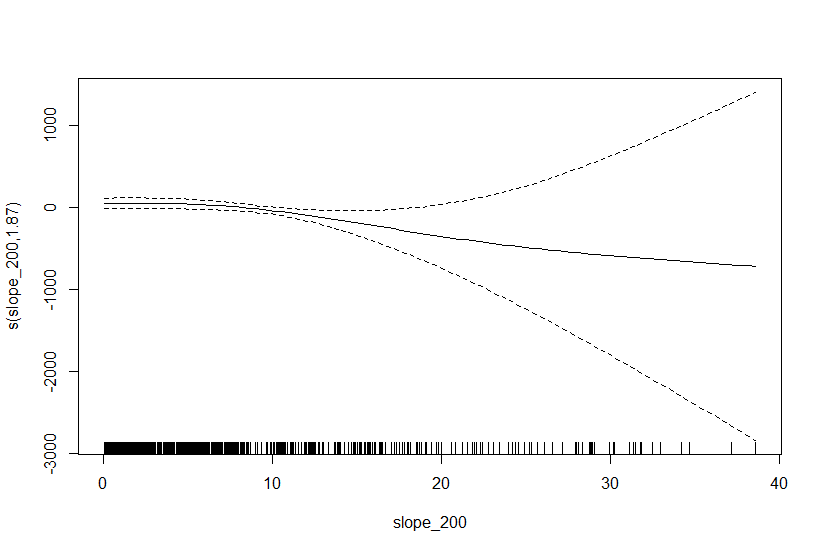
**

**
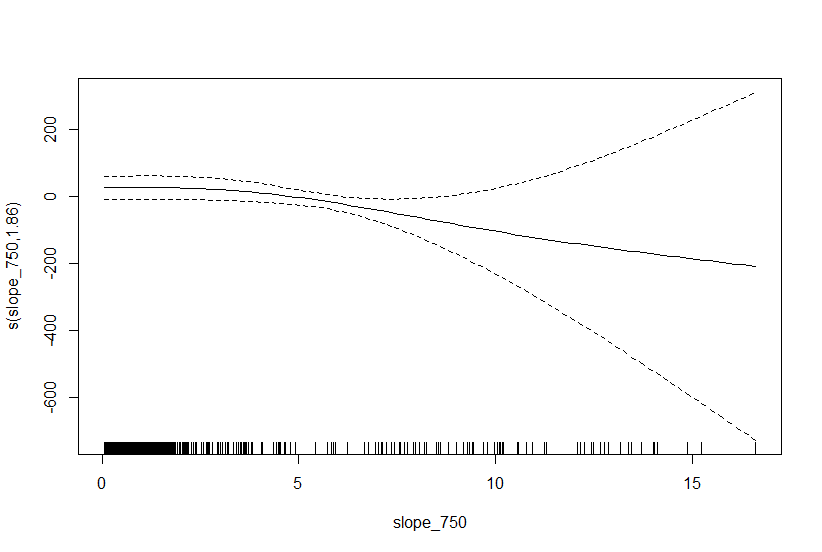
**

**c)**

**
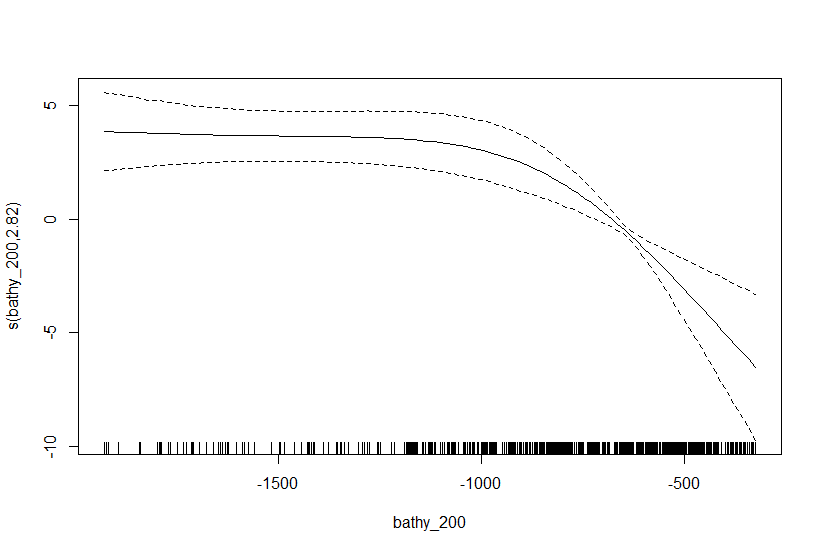
**

**
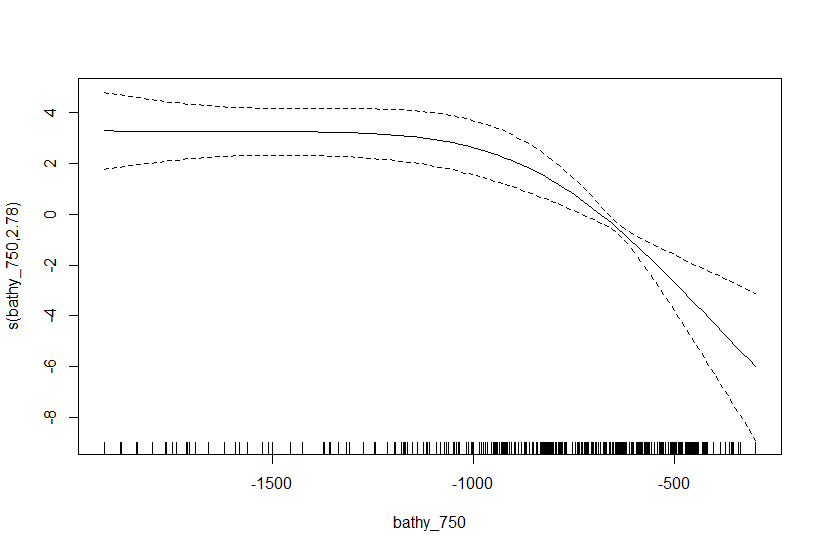
**

**
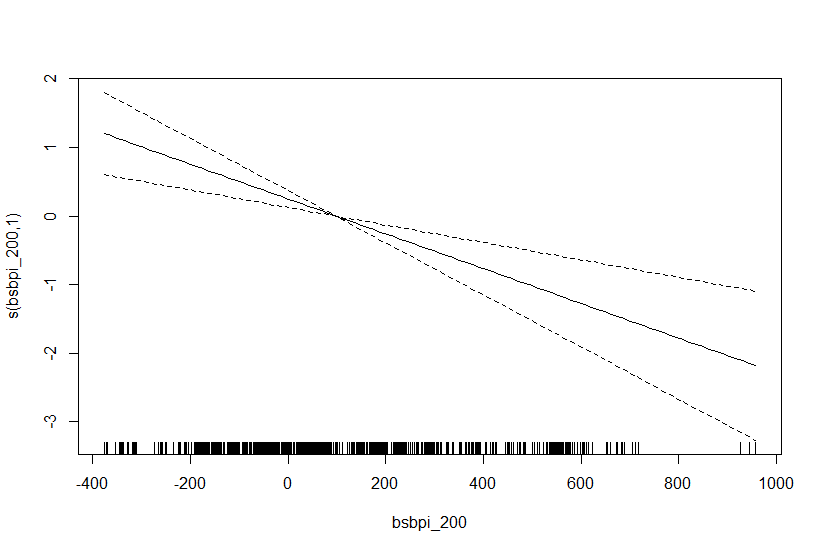
**

**
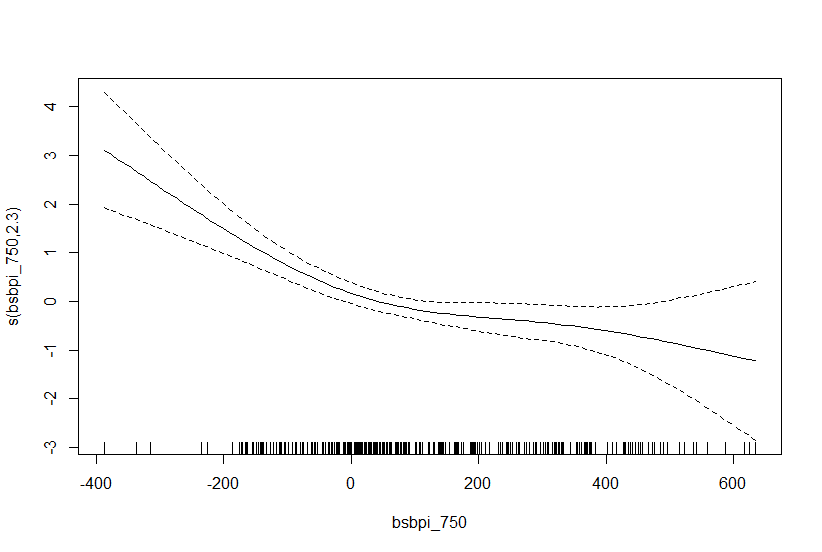
**

**
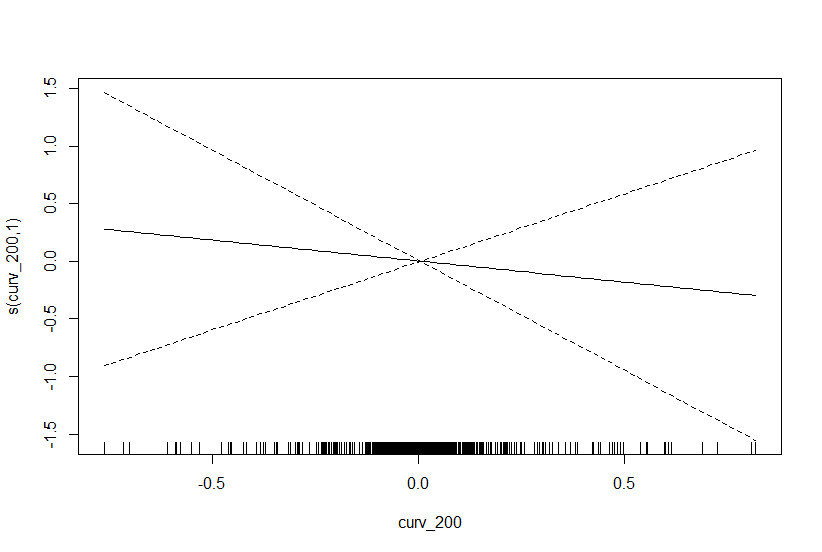
**

**
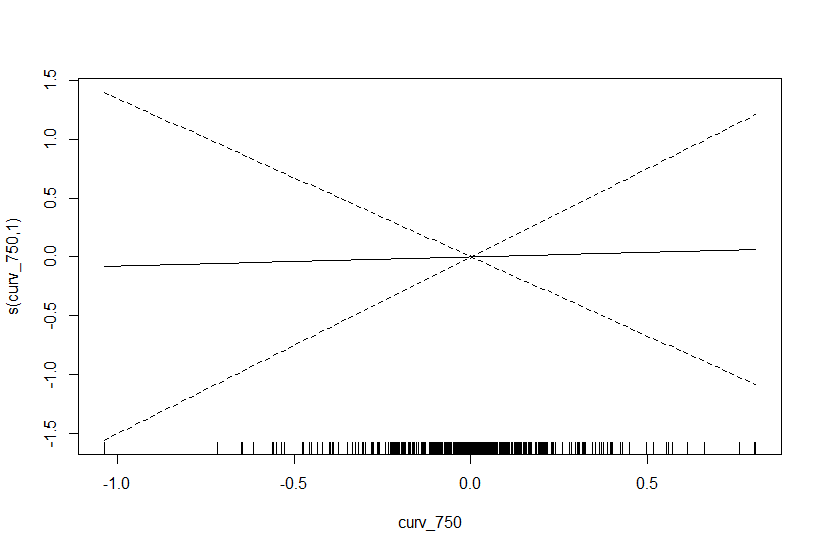
**

**
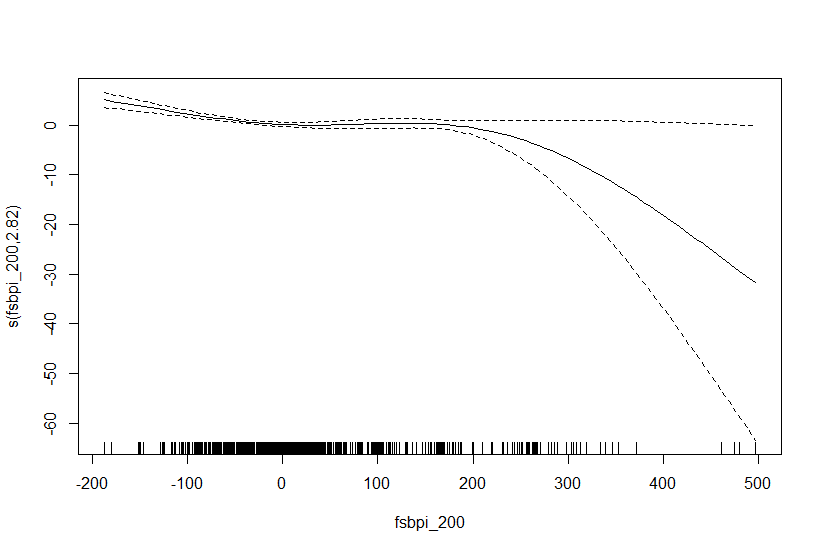
**

**
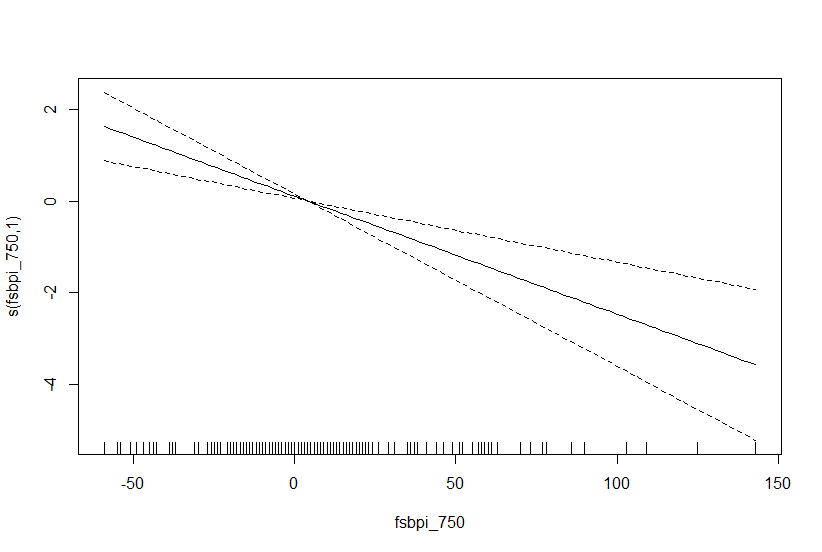
**

**
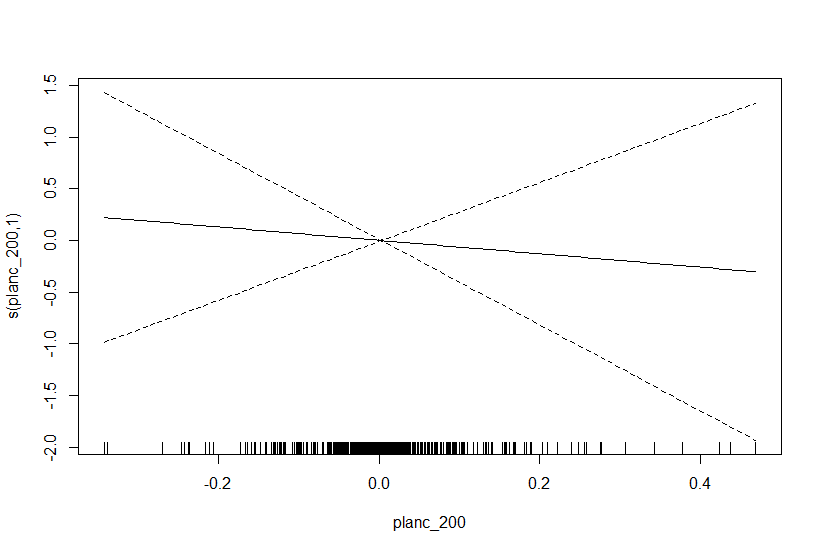
**

**
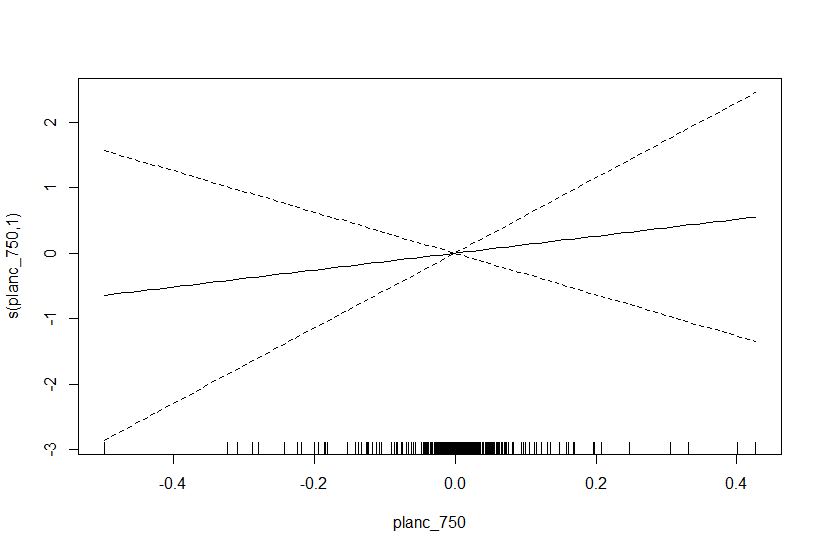
**

**
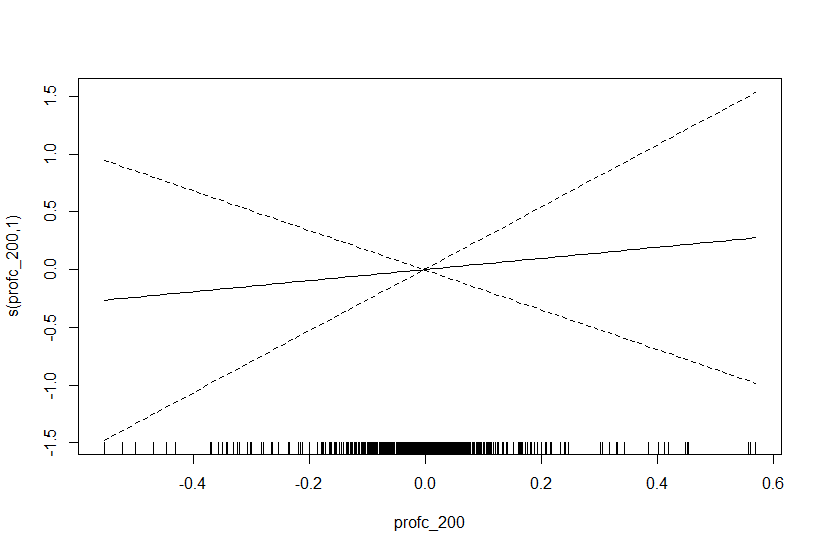
**

**
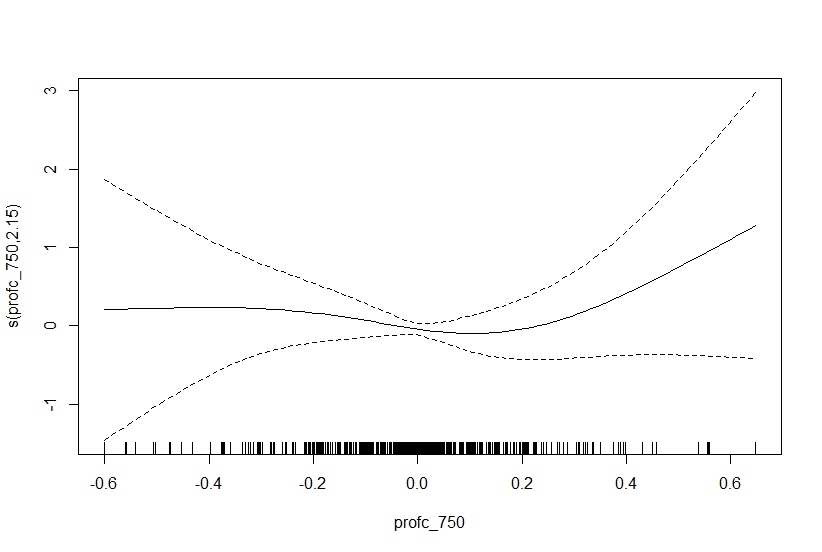
**

**
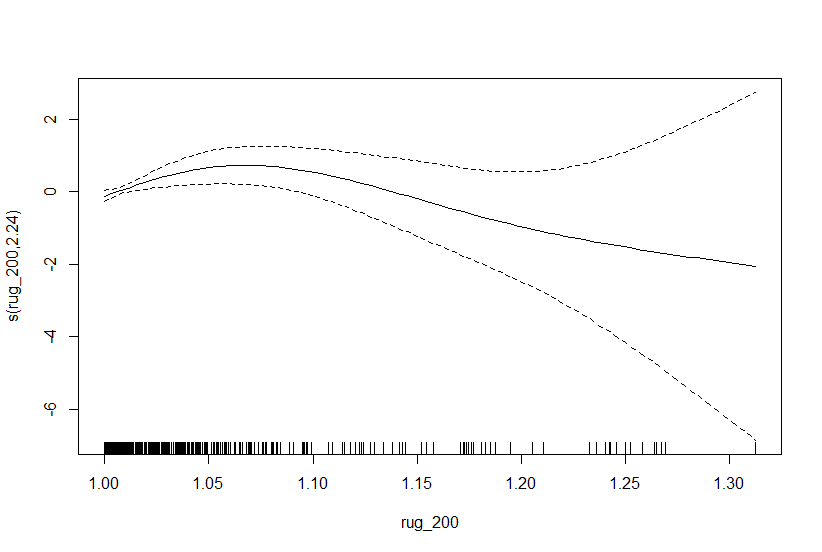
**

**
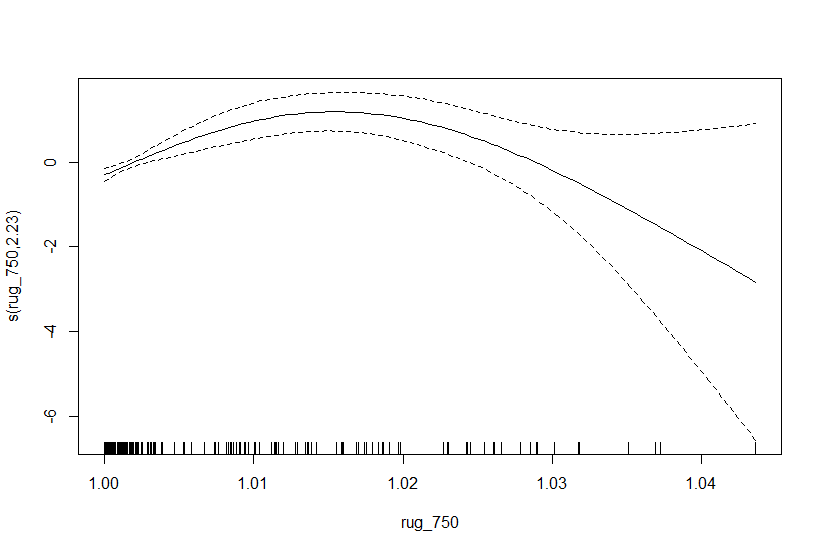
**

**
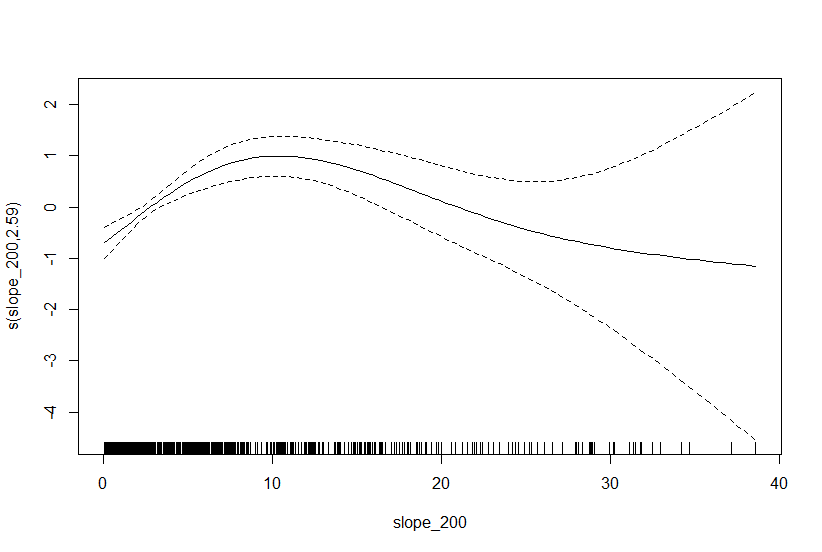
**

**
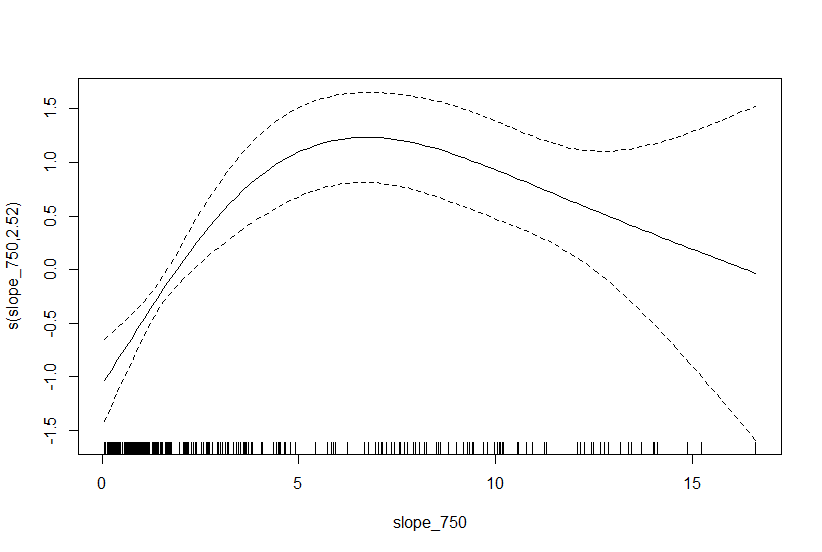
**

***Table B. Significance of smooth terms (P) and deviance explained (D, %).***

|  | a) | | b) | | c) | |
| --- | --- | --- | --- | --- | --- | --- |
| Variable | P | D (%) | P | D (%) | P | D (%) |
| Bathymetry (200m) | 0.01 | 3.7 | 0 | 54.6 | 0 | 27.9 |
| BPI broad (200m) | 0.01 | 0.538 | 0 | 34.7 | 0 | 3.3 |
| BPI fine (200m) | 0 | 1.96 | 0 | 26.4 | 0 | 12.7 |
| Curvature (200m) | 0.001 | 1.72 | 0.001 | 11.4 | **0.638** | 0.0392 |
| Plan curvature (200m) | 0 | 4.07 | 0.01 | 7.75 | **0.711** | 0.0246 |
| Profile curvature (200m) | **0.315** | 0.379 | 0 | 12.6 | **0.662** | 0.0337 |
| Rugosity (200m) | 0.001 | 2.09 | 0 | 13.5 | 0.01 | 1.93 |
| Slope (200m) | 0 | 6.17 | 0.05 | 12.5 | 0 | 5.17 |
| Bathymetry (750m) | 0.01 | 4.08 | 0 | 54.2 | 0 | 26 |
| BPI broad (750) | 0 | 6.17 | 0 | 26.6 | 0 | 6.48 |
| BPI fine (750m) | 0.01 | 1.15 | 0.001 | 13.9 | 0 | 3.74 |
| Curvature (750m) | **0.41** | 0.406 | 0.01 | 6.98 | **0.913** | 0.00221 |
| Plan curvature (750m) | **0.598** | 0.036 | **0.301** | 1.86 | **0.561** | 0.0592 |
| Profile curvature (750m) | **0.148** | 0.764 | 0.01 | 7.8 | **0.295** | 0.686 |
| Rugosity (750) | 0 | 1.5 | 0.001 | 10 | 0 | 5.03 |
| Slope (750) | 0 | 1.47 | 0.05 | 11.6 | 0 | 7.7 |

a) scleractinian cold-water coral reef; b) *Pheronema carpenteri* aggregations; c) *Syringammina fragilissima* aggregations. Values in bold indicates where p>0.05.

**Text B.**

Table B contains predictive capacity of each variable (described as % deviance from the no relationship 0-line). BPI broad (750m) and slope (200m) displayed the greatest predictive capacity for SclerReef presence (6.17%) followed by plan curvature (200m) (4.07%). Plan curvature (750m) displayed the least predictive capacity (0.036%) followed by profile curvature (200m) (0.379%). Dredge results indicated that a global model inclusive of BPIfine (200m), plan curvature (200m), BPIfine (750m), bathymetry (200m), BPIbroad (750m), curvature (200m), slope (200m), rugosity (200m), rugosity (750m), would yield the highest AICc score (AICc 640.6) confirming correct exclusion of correlated variables by their predictive capacity.

GAMs built with individual variables against the presence of SclerReef found the relationship between habitat occurrence and all variables to be significant except for profile curvature (750m) (p=0.148), curvature (750m) (p=0.41), profile curvature (200m) (p=0.315) and plan curvature (750m) (p=0.598) (column a in Table B), which were excluded from the final MaxEnt model.

Column b (Table B) lists the significance of the relationship of each individual variable with PcAggs occurrence. Plan curvature (750m) was the only variable that did not show a significant relationship (p=0.301) and was excluded from the final MaxEnt model. Bathymetry (200m) displayed the greatest predictive capacity (54.6%), closely followed by bathymetry (750m) (54.2%), then BPI broad (200m) (34.7%) (Table B). Plan curvature (750m) displayed the least predictive capacity (1.86%). Dredge results indicated that a global model inclusive of BPIfine (200m), plan curvature (200m), BPIfine (750m), bathymetry (200m), BPIbroad (200m), profile curvature (200m), rugosity (200m), slope (200m), profile curvature (750), slope (750) would yield the highest AICc score when correlates have been removed (AICc 105.7).

Profile curvature (750m) (p=0.295), plan curvature (750m) (p=0.561), curvature (200m) (p=0.638), profile curvature (200m) (p=0.662), plan curvature (200m) (p=0.711) and curvature (750m) (p=0.913) had no significant relationship with SfAggs occurrence (column c in Table B) and were excluded from the final MaxEnt model. Bathymetry (200m) displayed the greatest predictive capacity (27.9%) for SfAggs occurrence, followed by BPI fine (200m) (12.7%) (Table B2). Curvature (750m) displayed the least predictive capacity (0.002%). Dredge results indicated that a global model inclusive of BPI fine (200m), BPI fine (750m), bathymetry (200m), BPI broad (750m), slope (200m), rugosity (750m) and slope (750m) would yield the highest AICc score when correlates have been removed (AICc 328.03).

**Table C. Variable combinations.**

| a) | b) | c) |
| --- | --- | --- |
| BPI fine (200m) | BPIfine (200m) | BPI fine (200m) |
| Plan curvature (200m) | Plan curvature (200m) | BPI fine (750m) |
| BPI fine (750m) | BPI fine (750m) | Bathymetry (200m) |
| Bathymetry (200m) | Bathymetry (200m) | BPI broad (750m) |
| BPI broad (750m) | BPI broad (200m) | Slope (200m) |
| Curvature (200m) | Profile curvature (200m) | Rugosity (750m) |
| Slope (200m) | Rugosity (200m) | Slope (750m) |
| Rugosity (200m) | Slope (200m) | Biogeography |
| Rugosity (750m) | Profile curvature (750m) |  |
| Biogeography | Slope (750m) |  |
|  | Biogeography |  |

Final variable combinations used in MaxEnt models for a) scleractinian cold-water coral reef, b) *Pheronema carpenteri* aggregations; c) *Syringammina fragilissima* aggregations.

**S2 File References**

1. R Development Core Team. *R: A Language and Environment for Statistical Computing.* R Foundation for statistical Computing. 2011.
2. Wood SN. Fast stable restricted maximum likelihood and marginal likelihood estimation of semiparametric generalized linear models. Journal of the Royal Statistical Society (B). 2011; 73: 3-36
3. Kim YJ, Gu C. Smoothing spline Gaussian regression: more scalable computation via efficient approximation. Journal of the Royal Statistical Society B. 2004; 66: 337-356.
4. Barton K. *MuMIn: multi-model inference.* In: R package version >=2.12.0. 2012.
