# Supplementary material for: The Influence of Data Resolution on Predicted Distribution and Estimates of Extent of Current Protection of Three ‘Listed’ Deep-Sea Habitats
Source: PLoS One. 2015 Oct 23;10(10):e0140061. doi: 10.1371/journal.pone.0140061 (PMC4619891; doi:10.1371/journal.pone.0140061)

**SUPPORTING INFORMATION**

**The influence of data resolution on predicted distribution and estimates of extent of current protection of three ‘listed’ deep-sea habitats**

Lauren K. Ross, Rebecca E. Ross, Heather A. Stewart and Kerry L. Howell

**S3 File - Modelling results**

**Table A.** **MaxEnt percent contribution estimates.**

| a) | | b) | | c) | |
| --- | --- | --- | --- | --- | --- |
| Variable | % | Variable | % | Variable | % |
| Rugosity (200m) | 46 | Bathymetry (200m) | 67 | Bathymetry (200m) | 69 |
| Bathymetry (200m) | 28 | Rugosity (200m) | 23 | Biogeography | 18 |
| Biogeography | 17 | Biogeography | 4.7 | BPI fine (750m) | 5 |
| BPI fine (200m) | 3.3 | Slope (750m) | 1.8 | BPI fine (200m) | 3.9 |
| Curvature (200m) | 2.6 | BPI broad (200m) | 1.7 | BPI broad (750m) | 2.1 |
| Plan curvature (200m) | 1.4 | Slope (200m) | 1.5 | Rugosity (750m) | 2.1 |
| Slope (200m) | 0.8 | Plan curvature (200m) | 0 | Slope (200m) | 0.3 |
| Rugosity (750m) | 0.7 | Profile curvature (750m) | 0 | Slope (750m) | 0 |
| BPI fine (750m) | 0.5 | BPI fine (750m) | 0 |  |  |
| BPI broad (750m) | 0.3 | BPI fine (200m) | 0 |  |  |
|  |  | Profile curvature (200m) | 0 |  |  |

MaxEnt percent contribution estimates (%) for each variable towards final distribution models for: a) scleractinian cold-water coral reef; b) *Pheronema carpenteri* aggregations, and c) *Syringammina fragilissima* aggregations.

**Figure A.** **Confidence maps to support model predictions.** Standard deviations of predicted presence probabilities from the ten training/test partitioned models for a) scleractinian cold-water coral reef, b) *Pheronema carpenteri* aggregations, and c) *Syringammina fragilissima* aggregations. Low and high SD values in each are as follows: a) low SD 0.01-0.04, high SD 0.04-0.20; b) low SD 0.00-0.05, high SD 0.05-0.29; c) low SD 0.01-0.06, high SD 0.06-0.19. Maps are projected in Albers Equal Area Conic with modified standard parallels (parallel 1 = 50.2˚, parallel 2 = 58).


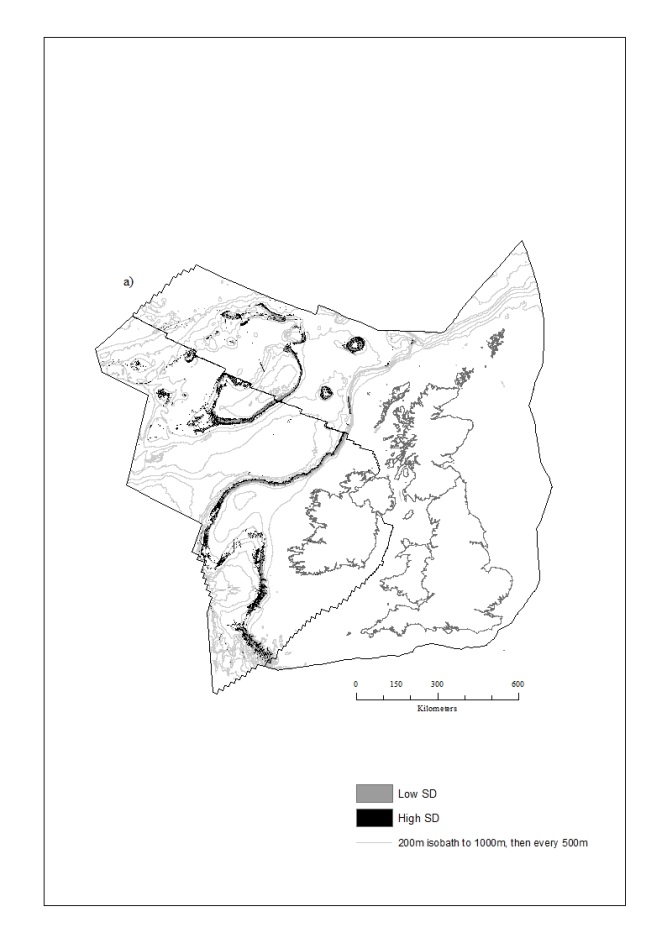

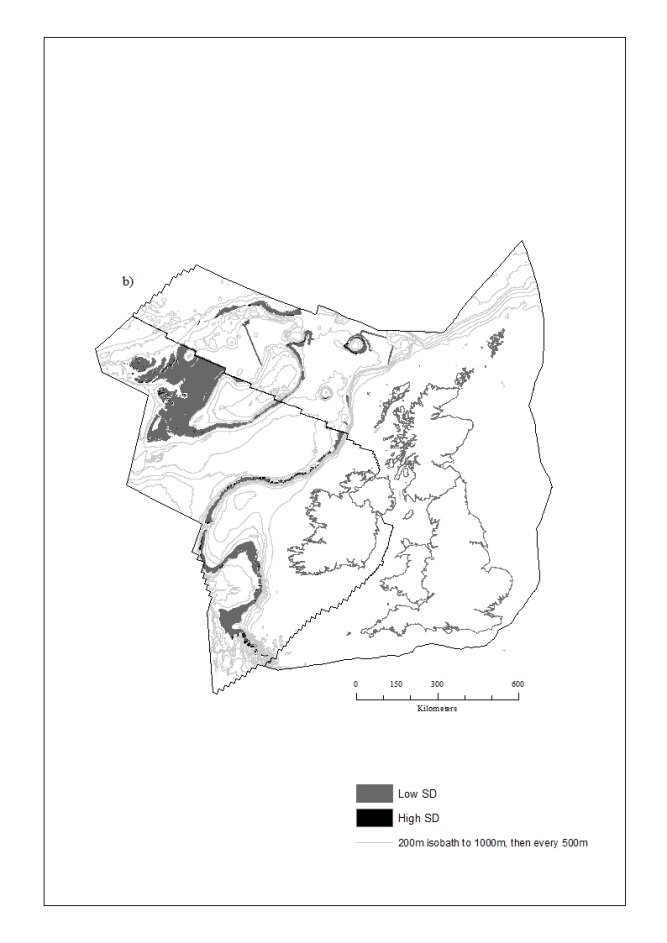

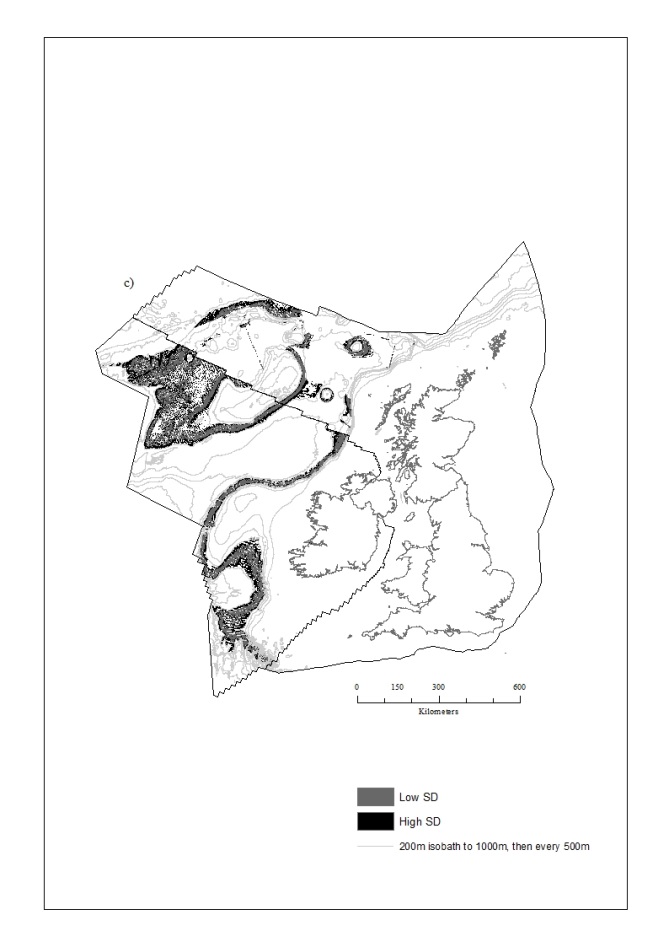

Supplement: S3 File — (DOC) [file pone.0140061.s003.doc]
